# Secretome profiling of PC3/nKR cells, a novel highly migrating prostate cancer subline derived from PC3 cells

**DOI:** 10.1371/journal.pone.0220807

**Published:** 2019-08-12

**Authors:** Ju Mi Jeon, Oh Kwang Kwon, Ann-Yae Na, Eun Ji Sung, Il Je Cho, Mirae Kim, Sung Su Yea, So Young Chun, Jun Hyung Lee, Yun-Sok Ha, Tae Gyun Kwon, Sangkyu Lee

**Affiliations:** 1 BK21 Plus KNU Multi-Omics Based Creative Drug Research Team, College of Pharmacy, Research Institute of Pharmaceutical Sciences, Kyungpook National University, Daegu, Republic of Korea; 2 College of Korean Medicine, Daegu Haany University, Gyeongsan, Gyeongsangbuk-do, Republic of Korea; 3 Department of Biochemistry, College of Medicine, Inje University, Busan, Republic of Korea; 4 Joint Institute for Regenerative Medicine, Kyungpook National University Hospital, Daegu, Republic of Korea; 5 Department of Urology, School of Medicine, Kyungpook National University, Daegu, Republic of Korea; 6 Department of Urology, Kyungpook National University Hospital, Daegu, Republic of Korea; Morehouse School of Medicine, UNITED STATES

## Abstract

Prostate cancer (PCa) is the most common cancer among men worldwide. Most PCa cases are not fatal; however, the outlook is poor when PCa spreads to another organ. Bone is the target organ in about 80% of patients who experience metastasis from a primary PCa tumor. In the present study, we characterized the secretome of PC3/nKR cells, which are a new subline of PC3 cells that were originally isolated from nude mice that were implanted with PC3 cells without anti-natural killer (NK) cell treatment. Wound healing and Transwell assays revealed that PC3/nKR cells had increased migratory and invasive activities in addition to a higher resistance to NK cells-induced cytotoxicity as compared to PC3 cells. We quantitatively profiled the secreted proteins of PC3/nKR and PC3 cells by liquid chromatography-tandem mass spectrometry analysis coupled with 2-plex tandem mass tag labeling. In total, 598 secretory proteins were identified, and 561 proteins were quantified, among which 45 proteins were secreted more and 40 proteins were secreted less by PC3/nKR cells than by PC3 cells. For validation, the adapter molecule crk, serpin B3, and cystatin-M were analyzed by western blotting. PC3/nKR cells showed the selective secretion of NKG2D ligand 2, HLA-A, and IL-6, which may contribute to their NK cell-mediated cytotoxicity resistance, and had a high secretion of crk protein, which may contribute to their high migration and invasion properties. Based on our secretome analysis, we propose that PC3/nKR cells represent a new cell system for studying the metastasis and progression of PCa.

## Introduction

Prostate cancer (PCa) is the most common cancer among men across the world. The American Cancer Society estimated that there were 161.360 new cases and 26,730 deaths from PCa in the United States during 2017 [1]. Although in many patients PCa progresses so slowly that it never causes a clinical problem, PCa can spread to other tissues, especially to bone [2]. While PCa is a serious disease, most diagnosed cases of PCa do not result in fatality. The 5-year relative survival rate of PCa diagnosed at a local or regional stage approaches 100% [1].

Unfortunately, once PCa has spread to the lymph nodes and bones, the outlook is poor. The 5-year survival rate for metastatic cancer is one-third of that for localized disease [3]. Statistically, 25% of men with PCa worldwide develop metastatic disease and the 5-year survival of patients with metastasis to a distant site is significantly reduced to 29% [4, 5]. Bone metastasis is a major cause of quality of life impairment and death among patients with metastatic PCa [6]. Among 3,857 patients presenting with metastatic PCa between 1991–2009, 2.8%, 80.2%, 6.1%, and 10.9% of patients had lymph node, bone, visceral, and bone plus visceral metastasis at diagnosis, respectively [7].

To study the metastasis of PCa to bone, the PC3 cell line is mainly used as a classical human prostatic carcinoma cell line [8]. It has a greatly reduced dependence upon serum for growth when compared to normal prostatic epithelial cells and does not respond to androgens, glucocorticoids, or epidermal/fibroblastic growth factors. Moreover, various cell lines derived from PC3 cells have been isolated after the injection of PC3 cells into nude mice for the detailed analysis of PCa metastasis [9]. For example, the PC3M cell line, which is a subline of PC3, was isolated from a PC3-induced mouse tumor [10]. PC3M-LN4 cells were harvested after the repeated injection of PC3M cells into the prostates of athymic mice, and this subline has a higher incidence of lung metastasis and bone metastasis than PC3M [9]. In addition, the PC3/nKR cell line, which is resistant to cytotoxicity mediated by natural killer (NK) cells, was isolated from mammary tumor xenograft studies in which PC3 was implanted into nude mice and found to be tumorigenic in the early 2000s [11].

The secretome is the total protein released by a cell, tissue, or organism through various mechanisms and is mainly involved in the cell-cell and tissue-tissue communications required for normal physiological functions [12, 13]. The secreted proteins provide information regarding the physiological condition of the source cells and contribute to diverse functions including immune regulation and pathological processes involved in cancer invasion and metastasis [14]. Characterizing the cancer cell secretome is important for bettering our understanding of molecular oncology [15]. As such, our previous studies have reported candidates that can be used for prostate cancer diagnosis and metastatic prediction through secretory protein analysis [16].

In this study, we investigated the secreted proteins in the conditioned media of the PC3 and PC3/nKR cell lines using a comparative proteomics approach to identify the molecular mechanisms related to the migration of PC3/nKR cells. Among 598 detected proteins, 560 proteins were quantified and 45 proteins were secreted in a higher amount by PC3/nKR cells as compared with PC3 cells. Our study showed that PC3/nKR cells are a new cell subline with a higher migration activity and increased NK cells resistance as compared with PC3 cells. These novel highly malignant tumor cells can be applied to study the mechanisms of PCa metastasis. We also identified secreted factors that may contribute to the high NK cells resistance and migration properties of PC3/nKR cells through a comparative secretome analysis.

## Materials and methods

### Cell culture

The human prostate cancer cell lines PC3 and PC3/nKR were obtained from the Korean Cell Line Bank (Seoul, Korea). PC3 and PC3/nKR cells were cultured in Roswell Park Memorial Institute 1640 medium (Pierce, Fremont, CA) supplemented with 10% fetal bovine serum (GE Healthcare, Little Chalfont, UK) and 1% penicillin and streptomycin (Gibco, Rockville, MD). All cells were maintained at 37°C with 5% CO_2_ in a humidified incubator.

### Protein extraction from conditioned medium

PC3 and PC3/nKR cells were grown to approximately ~85% confluence in 150-mm culture dishes. The cells were washed carefully with serum-free medium three times to avoid serum contamination and then incubated in serum-free medium for 24 h at 37°C. Conditioned media samples were centrifuged at 1,000 × *g* for 10 min to remove dead cells. The supernatant was concentrated using a concentrator filter (Millipore, Billerica, MA), and then incubated with protease inhibitor cocktail (Thermo Fisher Scientific, Waltham, MA). The protein concentrations of PC3 and PC3/nKR conditioned media samples were determined by the bicinchoninic acid protein assay. In addition, whole cellular proteins were isolated. The cells were washed twice with cold phosphate-buffered saline and lysed in radioimmunoprecipitation assay buffer supplemented with protease inhibitor cocktail. The cell lysates were collected, sonicated on ice, and centrifuged at 12,000 × *g* for 10 min at 4°C. The protein concentration was measured using the bicinchoninic acid assay. All protein samples were stored at –80°C until use.

### In-solution tryptic digestion and 2-plex tandem mass tag (TMT) labeling

For reduction and alkylation, 25 μg of protein samples were added to 200 mM Tris(2-carboxyethyl)phosphine and incubated at 55°C for 1 h. The proteins were alkylated with 60 mM iodoacetamide at room temperature for 30 min protected from light. Samples were subjected to acetone precipitation to purify proteins and incubated with 4 volumes of ice-cold acetone at −20°C for 4 h. Precipitated proteins were centrifuged at 14,000 × *g* for 10 min at 4°C and re-dissolved in 100 μL of 100 mM triethylammonium bicarbonate buffer (pH 8.5). Proteins were digested by trypsin (Promega, Madison, WI) at a ratio of 1:50 (trypsin:protein, w/w). For quantitative proteomics, each digested protein sample was labeled with an appropriate TMT reagent (Thermo Fisher Scientific) according to the protocol supplied by the manufacturer. Briefly, the PC3 and PC3/nKR peptide samples were labeled with reagents 127 and 128, respectively. To quench the TMT reaction, 8 μL of 5% hydroxylamine was added to each sample and incubated for 15 min at room temperature. All the labeled peptides were combined in equal amounts and dried using a speed vacuum system.

The peptide mixture was fractionated using a high-pH reverse-phase peptide fractionation kit (Thermo Fisher Scientific) according to the manufacturer’s procedure. Totally, 8 fractionated peptides were cleaned using ZipTip pipette tips (Millipore) to remove the salt and completely dried by a speed vacuum system followed by liquid chromatography-tandem mass spectrometry (LC-MS/MS).

### Nano LC-MS/MS analysis

The peptides were dissolved in solvent A (99.9% water with 0.1% formic acid) and analyzed using a Q Exactive Hybrid Quadrupole-Orbitrap Mass Spectrometer (Thermo Fisher Scientific) connected to an Easy-nLC 1000 liquid chromatograph (Thermo Fisher Scientific). Peptide separation was performed using a custom-made capillary column (13 cm length, 75 μm diameter) packed with ReproSil-Pur C18 3 μm beads (Dr. Maisch GmbH, Ammerbuch, Germany) with a linear gradient of 2–23% solvent B (99.9% acetonitrile with 0.1% formic acid) for 44 min and 23–90% solvent B for 5 min, followed by an isocratic flow of 30% solvent B for 11 min at a continuous flow rate of 200 nL/min.

The peptides were positively ionized using a nanospray ion source and mass spectrometry (MS) was conducted in top 15 data-dependent mode with the following settings: 2.2 kV ion spray voltage; 280°C ion capillary temperature, 300–1400 mass scan range for full MS and 120–2,000 scan range for MS/MS; MS resolution 70,000 for full MS and 17,500 for data-dependent MS/MS; collision energy 27 with high-energy collisional dissociation mode; MS/MS isolation window 1.5 m/z; and dynamic exclusion 25 s.

### MS/MS data analysis

The peptide and proteins were identified and quantified using MaxQuant 1.5 with human protein sequences from UniProt Swiss-Prot and TrEMBL (downloaded at http://www.uniprot.org; Proteome ID: UP000005640; 70630 protein sequence) as reference sequences [17, 18]. The search parameters were set as follows: the cleavage enzyme was trypsin/P and 2 miss cleavage; full MS and MS/MS tolerances were 10 ppm and 0.05 Da, respectively; fixed modification of carbamidomethylation on Cys, variable modification of oxidation on Met, and acetylation on protein *N*-termini were specified; proteins required a minimum of two peptides. For high quality proteomics results, MaxQuant search results were filtered using a peptide score of ≥40 and false discovery rate of ≤ 0.01, and reversed or potentially contaminating proteins were removed from the list of identified proteins. To calculate the ratio of TMT-labeled proteins between PC3 and PC3/nKR, the reporter ions were computed for 2-plex TMT labeling at peptide N-termini and lysine. Other parameters were set to default. The mass spectrometry proteomics data have been deposited to the ProteomeXchange Consortium via the PRIDE [19] partner repository with the dataset identifier PXD008184.

For the comparison of secretory proteins from database sets with our data, sets of secretory protein predicted using the majority decision-based method were downloaded from The Human Protein Atlas (https://www.proteinatlas.org/) and Plasma Proteome Database (http://www.plasmaproteomedatabase.org/) [20, 21]. Ensemble IDs from database sets were transferred to Uniprot ID using the Retrieve/ID mapping tool at Uniprot. Protein-protein interactions were extracted from the STRING database [22].

### Protein annotation and pathway enrichment

Gene ontology (GO) annotations of the proteome were derived from the UniProt-GOA database (http://www.ebi.ac.uk/GOA/). First, acetylated peptide IDs were converted to UniProt IDs and then mapped to GO IDs by protein ID. If identified acetylation substrates were not annotated by the UniProt-GOA database, the InterProScan software was used to annotate the protein’s GO function based on the protein sequence alignment method. Acetylated proteins were further classified by GO annotation based on three categories: biological process, cellular component, and molecular function. The Kyoto Encyclopedia of Genes and Genomes (KEGG) database was used to annotate the protein pathways. First, the KEGG automatic annotation server was used to annotate the protein’s KEGG database description. Then, annotation results were mapped onto the KEGG pathway database using the KEGG online tool Mapper. Domain annotation was performed using InterProScan to search the InterPro database via their web-based interfaces and services. WoLF PSORT was used for subcellular localization predictions. The CORUM database was used to annotate protein complexes.

For GO enrichment analysis, a two-tailed Fisher’s exact test was performed to test the enrichment of the differentially expressed protein against all identified proteins from each category. Correction for multiple hypothesis testing was employed using standard false discovery rate control methods. The GO with a corrected p-value < 0.05 is considered significant. The KEGG database was used to identify enriched pathways by a two-tailed Fisher’s exact test to assess the enrichment of the differentially expressed proteins against all identified proteins. Correction for multiple hypothesis testing was performed using standard false discovery rate control methods. Pathways with a corrected *p*-value < 0.05 were considered significant. These pathways were classified into hierarchical categories according to the KEGG website.

For analysis of protein-protein networks, STRING was used with the different expressed proteins [22]. Proteins with an interaction score of ≤0.4 and uncoupled proteins were removed from the protein-protein network. The other parameters in STRING were set to default values.

### Wound healing and Transwell invasion assays

To observe the migratory capacities of PC3 and PC3/nKR cells, 0.6 × 10^6^ cells were seeded on each well of a 6-well plate (Corning, NY, USA) and incubated until they reached ~90% confluence. The monolayer cells were artificially scratched with a sterile 1,000-μL tip and washed with phosphate-buffered saline twice to remove cell debris. Next, serum-free Roswell Park Memorial Institute 1640 media was added and then wounded cells were observed under a light microscope (Olympus, Tokyo, Japan) at intervals of 12 h and imaged via a Las EZ microscope camera (Leica Microsystems, Wetzlar, Germany) coupled with automatically measurement software (CellProfiler-2, v2.0, Broad Institute). To examine the effects of the proteins secreted by PC3 and PC3/nKR cells on the migration of the other cell line, conditioned media (CM) from each cell line were exchanged with each other and then incubated for 24 h. Migration ability in cells was compared by the value of relative migration defined as the reciprocal of the relative distance when the cell is restored after scratching.

A Transwell invasion assay was performed to detect the invasiveness of PC3 and PC3/nKR cells. 200 μg/mL Matrigel matrix (Corning) was added into permeable supports with 8-μm pores (Corning) immediately before use. Next, each cell suspension was seeded (0.12 × 10^6^ cells) in the upper chamber and media containing 10% fetal bovine serum was added into the lower chamber. After 24 h, non-invaded cells on the upper chamber were carefully rubbed off using a cotton swab and then the cells that had invaded through the Matrigel matrix were stained with 0.5% crystal violet (Sigma-Aldrich, St. Louis, MO) and observed via light microscopy. Lastly, the optical density of the membrane with the attached invaded cells was measured at 570 nm.

### Cytotoxicity

NK cell-mediated cytotoxicity was measured by the calcein-acetoxymethyl release assay, which has a sensitivity similar to that of the traditional ^51^Cr release assay as described previously [23, 24]. Briefly, 5 × 10^3^ target cells/well were mixed with NK effector cells at a 10:1 effector:target ratio in pentaplicate and incubated for 4 h at 37°C in IL-2-free medium in round-bottomed 96-well plates. Target cells including PC3 and PC3/nKR cells were labeled with 10 μM calcein-acetoxymethyl (Molecular Probes, Eugene, OR), washed, and used for co-culture with human NK cells. After 4 h of co-culture, 100 μL aliquots of culture supernatants were transferred to a 96-well black plate (Nalge Nunc International, Rochester, NY) and arbitrary fluorescent units (AFU) were read using a SpectraMAX Gemini XS plate reader (Molecular Devices, San Jose, CA) at 485 nm excitation and 538 nm emission. The percentage of specific lysis from the pentaplicate experiments was calculated using the following equation: % specific lysis = ((AFU mean experimental release − AFU mean spontaneous release) / (AFU mean maximal release − AFU mean spontaneous release)) × 100, where “AFU mean spontaneous release” is calcein release by target cells in the absence of NK cells and “AFU mean maximal release” is calcein release by target cells upon lysis by 2% Triton X-100.

### Western blotting

The secreted proteins and cell lysates of PC3 and PC3/nKR were prepared as previously described. The sample was loaded 10 μg respectively and separated via 10% sodium dodecyl sulfate polyacrylamide gel electrophoresis 120 V for 90 min and transferred to polyvinylidene fluoride blotting membranes (0.2 μm pore size, GE Healthcare) 90 V for 2 h. The membrane was blocked in 5% bovine serum albumin for 2 h and then incubated overnight with primary antibody at 4°C. Blots were washed in Tris-buffered saline containing 0.05% Tween 20 three times for 15 min each time. First antibodies (mouse-anti CRK #MA515891, rabbit-anti SERPINB3 #PA5-31891, rabbit-anti CystatinM #TA337639, rabbit-anti Follistatin #PA5-19787, mouse-anti ULBP2 #ab88645, rabbit-anti β-actin #4967, rabbit-anti α-tubulin #52866) were diluted 1:1000 each for detecting target proteins. The membrane was then incubated with a horseradish peroxidase-conjugated secondary antibody (either rabbit or mouse) at room temperature for 2 h. The blots were washed in Tris-buffered saline containing 0.05% Tween 20 three times again and visualized using ECL Prime western blotting detection reagent (GE Healthcare).

### Data and statistics

All the experiments were repeated at least three times and the data were expressed as mean values ± standard error. Statistical analyses were performed using SPSS Statistics version 21 (IBM, Armonk, NY) to determine significant differences. Significant values (*P <* 0.05) were represented in the Figures using asterisks (*P < 0.05, **P < 0.01, ***P < 0.001).

## Results

### The migration and invasion characteristics of PC3/nKR cells

**Fig 1:**
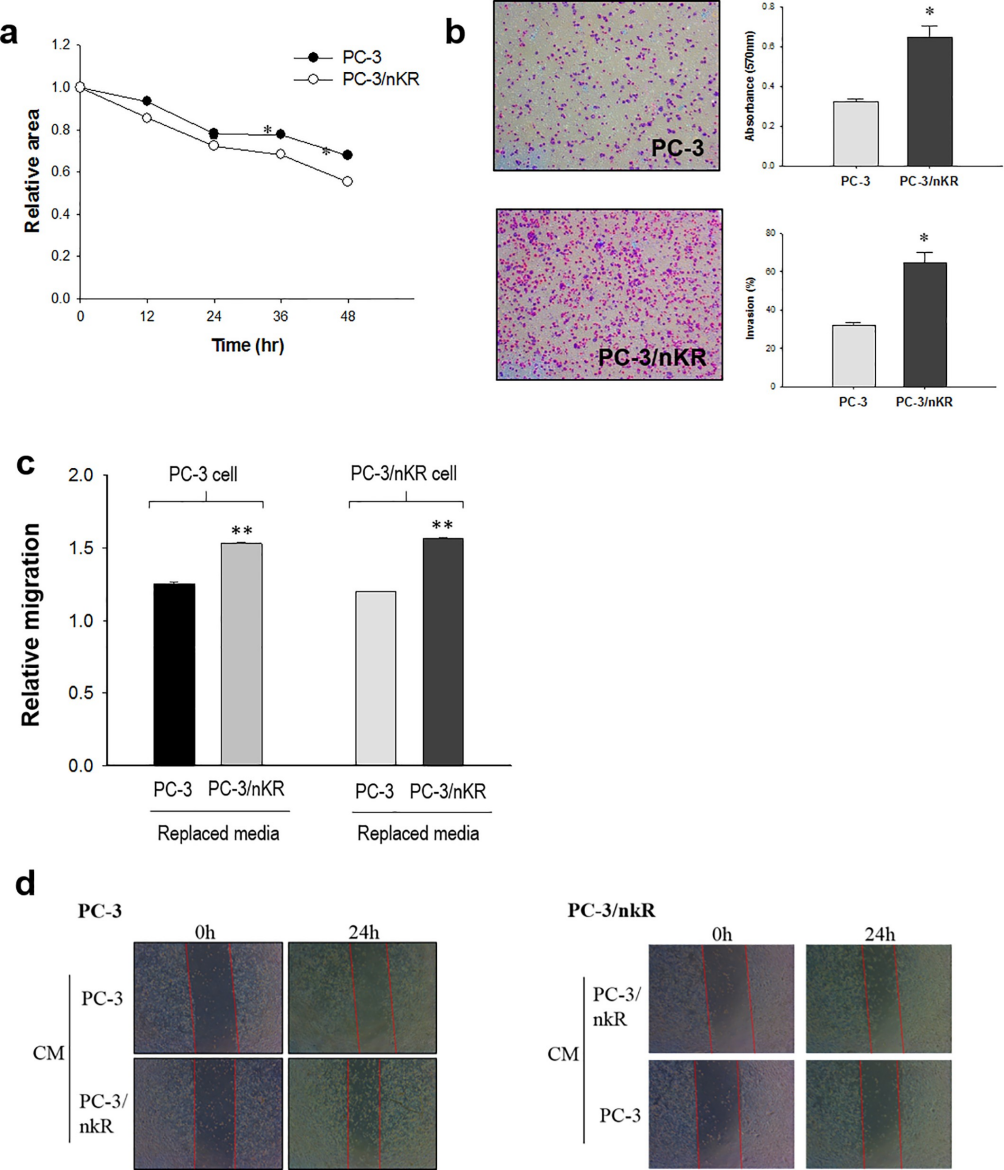
The characteristics of PC3 and PC3/nKR cells. (A) The relative wound area in wound scratch assays PC-3 and PC-3/nKR cells scratched by a tip were observed under light microscopy at intervals of 12 h and the results were normalized to those for PC-3 cells. (B) Representative images of cells that had invaded into a Transwell chamber are shown. (C) Effects of incubating PC-3/PC-3/nKR cells with the conditioned media (CM) from PC-3/nKR cells for 48 h, respectively. All the experiments were conducted in triplicate (**P < 0.01, ***P < 0.001). Relative migration is defined as the reciprocal of the relative distance when the cell is restored after scratching. (D) The representative pictures of wound healing assay for effect on condition media (CM) of PC-3/nKR on PC-3 cells and PC-3 on PC-3/nKR for 48 hrs, respectively.

PC3/nKR cells were originally isolated from nude mice that were implanted with PC3 cells without anti-NK treatment. According to an analysis of 100x pictures taken under light microscopy, we found that PC3/nKR and PC3 cells showed no detectable differences in cell morphology (**S1A Fig)**. To characterize the aggressiveness of PC3/nKR cells, their migratory and invasive activities were examined and compared to those of PC3 cells. A wound scratch assay was performed to compare the migration activities of the two cell lines. After creating a scratch wound, pictures were taken every 12 h for 48 h and we observed that PC3/nKR cells migrated further than PC3 cells (**S1B Fig**). The PC3/nKR cells had migrated significantly further than the PC3 cells after 48 h (**Fig 1A**). In the Transwell migration and invasion assay using a permeable chamber, the PC3/nKR cells showed significantly increased cell migration and a higher number of invaded cells as compared with the PC3 cells (**Fig 1B**). Moreover, the PC3/nKR cells showed less calcein release than PC3 cells when they were mixed with NK effector cells at a 10:1 effector:target ratio, which indicated that the PC3/nKR cells had a higher resistance to NK cell-mediated cytotoxicity than the PC3 cells (**S1C Fig**). Thus, these results indicated that PC3/nKR cells are more malignant than PC3 cells.

To evaluate the contribution of the secretome to the high migration and invasion activities of the PC3/nKR cells, CM were collected from cultures of PC3/nKR and PC3 cells and then incubated with the other cell line, and the effect on cell migration was evaluated (**Fig 1C and 1D**). PC3 cells cultured with the CM from PC3/nKR cells for 48 h showed increased migration, while PC3/nKR cells cultured with the CM from PC3 cells showed decreased migration. This result showed that specific factors secreted from PC3/nKR cells can stimulate the migration of PCa cells.

### Comparative proteomic profiling of CM from PCa cell lines

**Fig 2:**
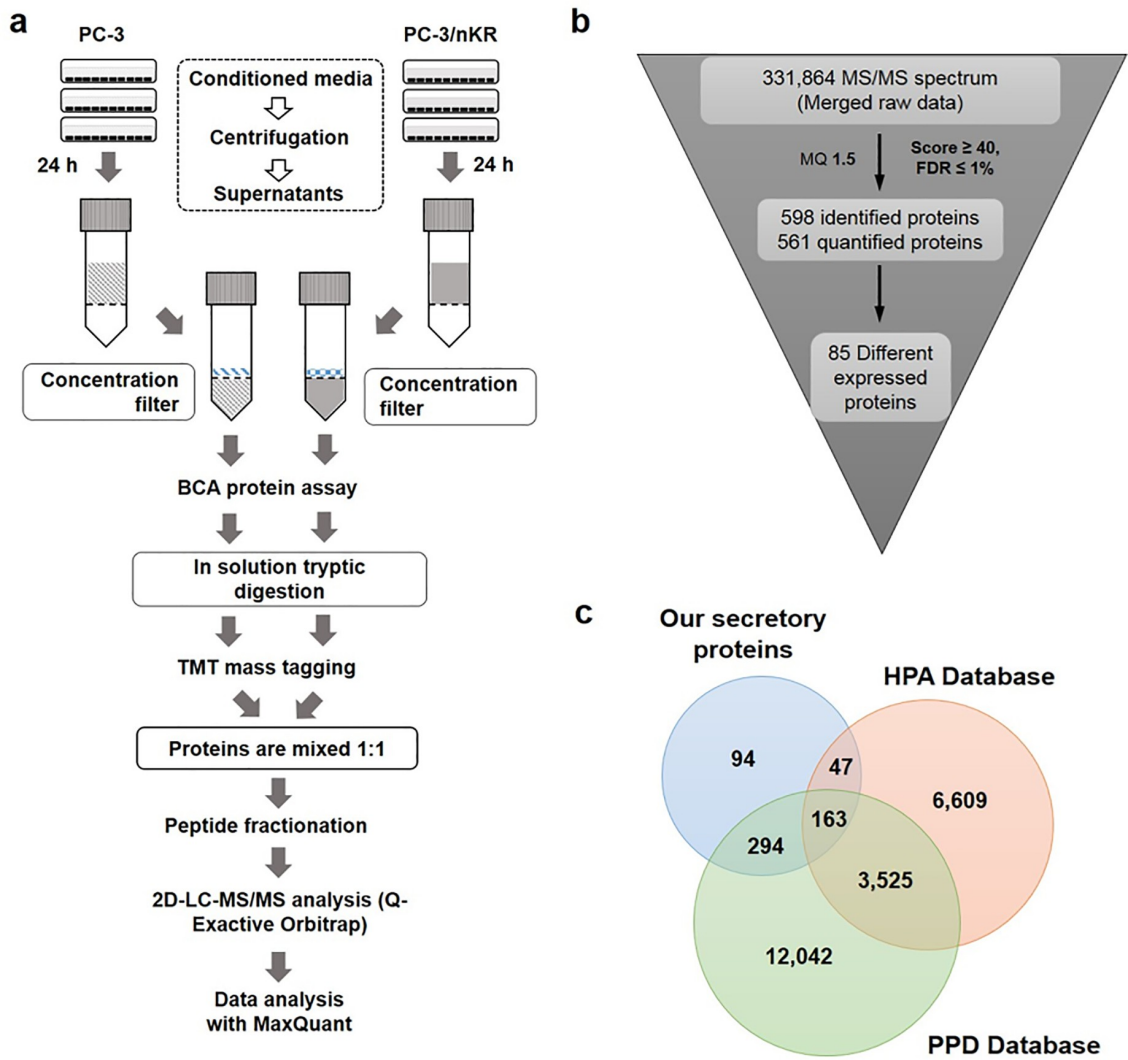
The experimental strategy for the quantitative analysis of the secretomes of PC3 and PC3/nKR cells. (A) The integrated workflow for the comparative proteomics analysis of proteins secreted from PC3 and PC3/nKR cells. (B) A schematic showing the processes for protein identification and quantification based on the results of the proteomic analysis. (C) A Venn diagram indicating that 94 of the 598 proteins that we identified were characterized as newly secretory based upon our results, as determined by comparison with The Human Protein Atlas and Plasma Proteome Database.

To identify factors stimulating the migration of PC3 cells, we quantitatively profiled the secreted proteins of PC3/nKR and PC3 cells by LC-MS/MS analysis with the 2-plex TMT labeling method (**Fig 2A**). Before proteomic analysis, to confirm the purity of the secreted proteins, we performed western blotting for α-tubulin and compared the results between the total cell lysates and CM of PC3 and PC3/nKR (**S2A Fig**). α-Tubulin was clearly detected in the cell extracts, but not in the CM. In addition, sodium dodecyl sulfate polyacrylamide gel electrophoresis with colloidal Coomassie blue staining showed that there were no major contaminating proteins such as albumin. Based on these results, the purity of the concentrated proteins was deemed to be suitable for MS-based secretome analysis.

In the LC-MS/MS analysis, 598 proteins secreted from PC3 and PC3/nKR cells were identified, among which 561 proteins (93.8%) were quantified based on the proteomic analysis (**Fig 2B, S1 Table**). The concordance of the identified proteins between a pair of technical replicates was identified as 89.2% similar (**S2B Fig)**. In addition, the intensities of the co-detected proteins showed a significant correlation with an *R* value of 0.9 or higher (**S2C Fig)**. Based on these results, our proteomics results were considered to have experimental significance. Among the quantified proteins, we identified 85 differentially expressed proteins, constituting 45 proteins that were secreted at higher levels by PC3/nKR cells (PC3/nKR *vs*. PC3 ratio >2) and 40 proteins that were secreted at lower levels by PC3/nKR cells (PC3/nKR *vs*. PC3 ratio <0.5) as compared to PC3 cells (**Tables 1 and 2**). To find new secreted proteins, the identified proteins were compared with The Human Protein Atlas (https://www.proteinatlas.org/) and Plasma Proteome Database (http://www.plasmaproteomedatabase.org/) [20, 21]. Among the 598 proteins we identified, 504 proteins were expected or identified to be secretory based on the information in these online databases; however, the other 94 proteins were newly identified as secretory from our results (**Fig 2C**).

**Table 1:** List of proteins secreted at higher levels by PC3/nKR cells than by PC3 cells.

Protein accession	Protein description	Ratio (PC3/nKR *vs* PC3)	Number of peptides	Type	Gene name
Q15828	Cystatin-M	8.69	2	Up	*CST6*
P19957	Elafin	5.627	3	Up	*PI3*
Q59FP8	Neogenin	5.278	2	Up	*NEO1*
P80188	Neutrophil gelatinase-associated lipocalin	5.073	5	Up	*LCN2*
B7Z5J4	Carboxypeptidase A4	4.769	2	Up	*CPA4*
E9PKG2	Low-density lipoprotein receptor-related protein 8	4.741	2	Up	*LRP8*
Q9GZL7	Ribosome biogenesis protein WDR12	4.738	3	Up	*WDR12*
Q5K684	Serpin B3; serpin B4	4.533	3	Up	*SERPINB3; B4*
O14672	Disintegrin and metalloproteinase domain-containing protein 10	4.439	3	Up	*ADAM10*
P03973	Antileukoproteinase	3.957	3	Up	*SLPI*
Q8WW12	PEST proteolytic signal-containing nuclear protein	3.642	2	Up	*PCNP*
Q92876	Kallikrein-6	3.521	2	Up	*KLK6*
Q8NBP7	Proprotein convertase subtilisin/kexin type 9	3.376	2	Up	*PCSK9*
Q99674	Cell growth regulator with EF hand domain protein 1	3.364	5	Up	*CGREF1*
P05198	Eukaryotic translation initiation factor 2 subunit 1	3.341	2	Up	*EIF2S1*
Q15459	Splicing factor 3A subunit 1	3.195	2	Up	*SF3A1*
B1AKC9	Ephrin type-B receptor 2	3.192	4	Up	*EPHB2*
Q13200	26S proteasome non-ATPase regulatory subunit 2	3.111	2	Up	*PSMD2*
K7EL68	Hsp90 co-chaperone Cdc37	3.107	3	Up	*CDC37*
G3V2V8	Epididymal secretory protein E1	3.056	6	Up	*NPC2*
P43121	Cell surface glycoprotein MUC18	2.975	2	Up	*MCAM*
P46108	Adapter molecule crk	2.748	2	Up	*CRK*
O75635	Serpin B7	2.648	4	Up	*SERPINB7*
C9J5B0	Interleukin-6	2.645	4	Up	*IL-6*
P17174	Aspartate aminotransferase, cytoplasmic	2.571	7	Up	*GOT1*
Q8NC51	Plasminogen activator inhibitor 1 RNA-binding protein	2.538	5	Up	*SERBP1*
D3DQB3	Testican-1	2.477	2	Up	*SPOCK1*
P30447	HLA class I histocompatibility antigen, A-23 α chain	2.436	6	Up	*HLA-;-H;-C*
Q5H9A7	Metalloproteinase inhibitor 1	2.428	6	Up	*TIMP1*
P19022	Cadherin-2	2.396	3	Up	*CDH2*
P42830	C-X-C motif chemokine 5	2.396	4	Up	*CXCL5*
Q92945	Far upstream element-binding protein 2	2.272	2	Up	*KHSRP*
P04062	Glucosylceramidase	2.261	6	Up	*GBA*
A0A087WZH7	Myristoylated alanine-rich C-kinase substrate	2.252	6	Up	*MARCKS*
K7EN15	Soluble calcium-activated nucleotidase 1	2.149	3	Up	*CANT1*
Q9BYC5	α-(1,6)-Fucosyltransferase	2.134	2	Up	*FUT8*
B8ZZU8	Transcription elongation factor B polypeptide 2	2.116	2	Up	*TCEB2*
P29279	Connective tissue growth factor	2.09	2	Up	*CTGF*
P12429	Annexin A3; annexin	2.076	7	Up	*ANXA3*
Q969H8	UPF0556 protein C19orf10	2.069	3	Up	*C19orf10*
Q9UNZ2	NSFL1 cofactor p47	2.066	7	Up	*NSFL1C*
F5H6X6	Neutral α-glucosidase AB	2.063	11	Up	*GANAB*
F8WEX7	Cholinesterase	2.062	2	Up	*BCHE*
P17931	Galectin-3	2.022	3	Up	*LGALS3*
A0A0C4DGH5	Cullin-associated NEDD8-dissociated protein 1	2.008	2	Up	*CAND1*

**Table 2:** List of proteins secreted less by PC3/nKR cells than by PC3 cells.

Protein accession	Protein description	Ratio (PC3/nKR *vs* PC3)	Number of peptides	Type	Gene name
Q1L6U9	Prostate-associated microseminoprotein	0.076	3	Down	*MSMP*
P05121	Plasminogen activator inhibitor 1	0.217	7	Down	*SERPINE1*
E5RHV6	CMP-*N*-acetylneuraminate-β-galactosamide-α-2,3-sialyltransferase 1	0.226	2	Down	*ST3GAL1*
Q5T0D2	UMP-CMP kinase	0.232	2	Down	*CMPK1*
P48444	Coatomer subunit delta	0.233	2	Down	*ARCN1*
Q06323	Proteasome activator complex subunit 1	0.266	3	Down	*PSME1*
Q9HC84	Mucin-5B	0.273	6	Down	*MUC5B*
Q16555	Dihydropyrimidinase-related protein 2	0.295	3	Down	*DPYSL2*
O15230	Laminin subunit α-5	0.305	16	Down	*LAMA5*
Q9BZM5	NKG2D ligand 2	0.319	3	Down	*ULBP2*
F8W1K5	Protein canopy homolog 2	0.335	2	Down	*CNPY2*
P09529	Inhibin β B chain	0.362	2	Down	*INHBB*
Q9H3G5	Probable serine carboxypeptidase CPVL	0.363	3	Down	*CPVL*
F8WAE5	Eukaryotic translation initiation factor 2A	0.368	2	Down	*EIF2A*
J3QRX2	Granulocyte colony-stimulating factor	0.37	2	Down	*CSF3*
Q9UBS4	DnaJ homolog subfamily B member 11	0.372	2	Down	*DNAJB11*
P28072	Proteasome subunit β type-6	0.375	2	Down	*PSMB6*
H0YM70	Proteasome activator complex subunit 2	0.393	2	Down	*PSME2*
Q13753	Laminin subunit γ-2	0.395	2	Down	*LAMC2*
Q32MZ4	Leucine-rich repeat flightless-interacting protein 1	0.403	2	Down	*LRRFIP1*
P13667	Protein disulfide-isomerase A4	0.411	12	Down	*PDIA4*
P27348	14-3-3 protein θ	0.413	2	Down	*YWHAQ*
P62805	Histone H4	0.417	2	Down	*HIST1H4A*
Q5T123	SH3 domain-binding glutamic acid-rich-like protein 3	0.424	2	Down	*SH3BGRL3*
Q13630	GDP-ʟ-fucose synthase	0.435	2	Down	*TSTA3*
P04155	Trefoil factor 1	0.445	3	Down	*TFF1*
P62937	Peptidyl-prolyl *cis*-*trans* isomerase A	0.451	7	Down	*PPIA*
P49006	MARCKS-related protein	0.452	3	Down	*MARCKSL1*
P13693	Translationally-controlled tumor protein; TPT1-like protein	0.454	3	Down	*TPT1*
P15018	Leukemia inhibitory factor	0.454	2	Down	*LIF*
P12956	X-ray repair cross-complementing protein 6	0.459	5	Down	*XRCC6*
P12109	Collagen α-1 (VI) chain	0.461	26	Down	*COL6A1*
Q8IWU5	Extracellular sulfatase Sulf-2	0.467	5	Down	*SULF2*
Q08380	Galectin-3-binding protein	0.467	8	Down	*LGALS3BP*
P02545	Prelamin-A/C; lamin-A/C	0.467	13	Down	*LMNA*
H0Y704	Zinc finger protein 185	0.467	2	Down	*ZNF185*
Q71UI9	Histone H2A.V	0.468	2	Down	*H2AFV*
Q9NTK5	Obg-like ATPase 1	0.478	2	Down	*OLA1*
P01036	Cystatin-S	0.495	2	Down	*CST4*
A0A0A0MSM0	Heat shock protein 105 kDa	0.495	3	Down	*HSPH1*

### Confirmation of the high secretion of specific proteins by PC3/nKR cells

**Fig 3:**
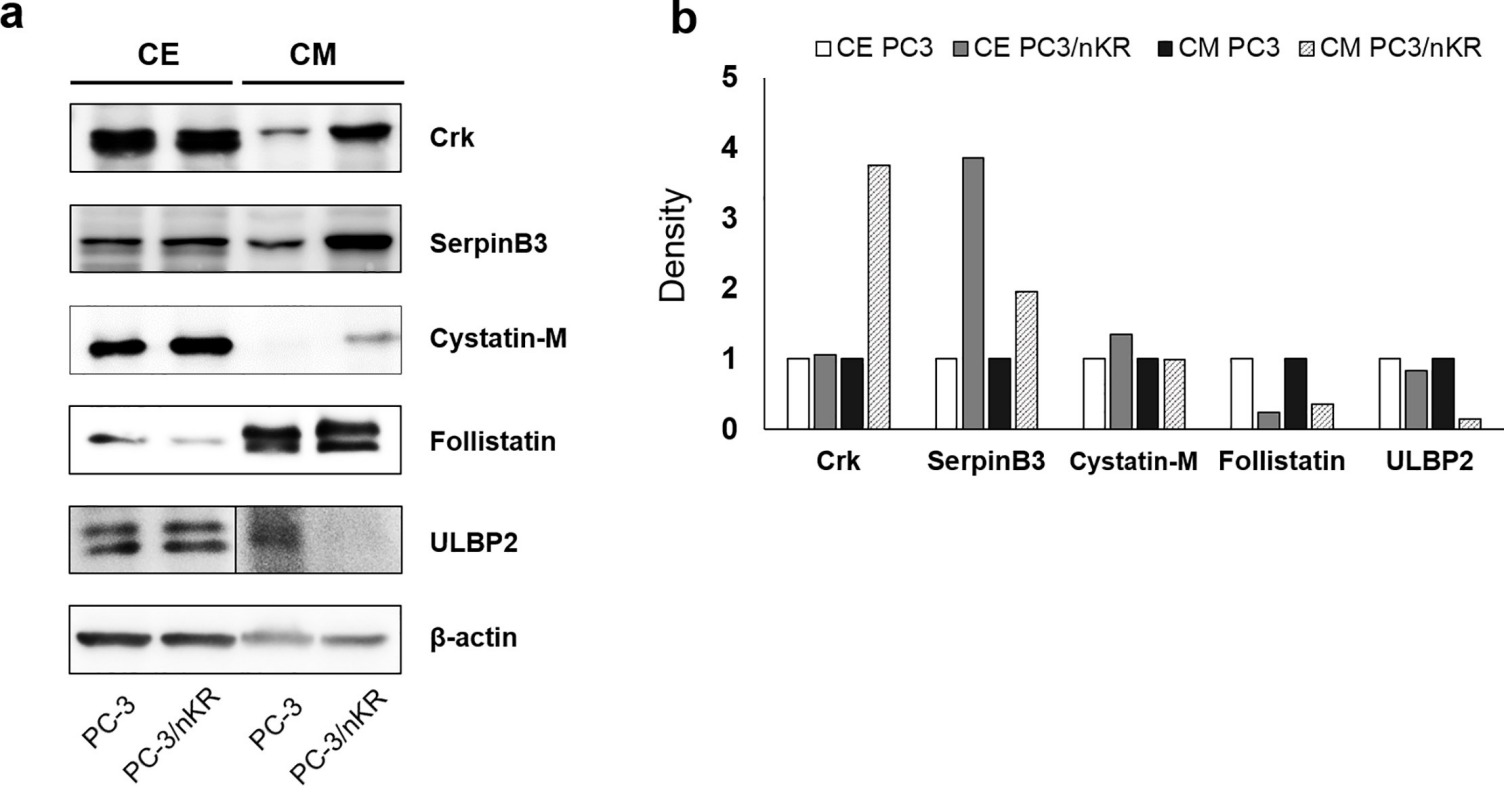
Validation of quantitative proteomics result using immunoblots. (A) Western blotting analysis of proteins with increased (Crk, SerpinB3, Cystatin-M), decreased (ULBP2), and unchanged secretion (Follistatin) in conditioned media (CM) and cell extract (CE) samples from PC3/nKR cells as compared with those from PC3 cells. (B) Densitometry results of Crk, SerpinB3, Cystatin-M, ULBP2 and Follistatin.

**Fig 4:**
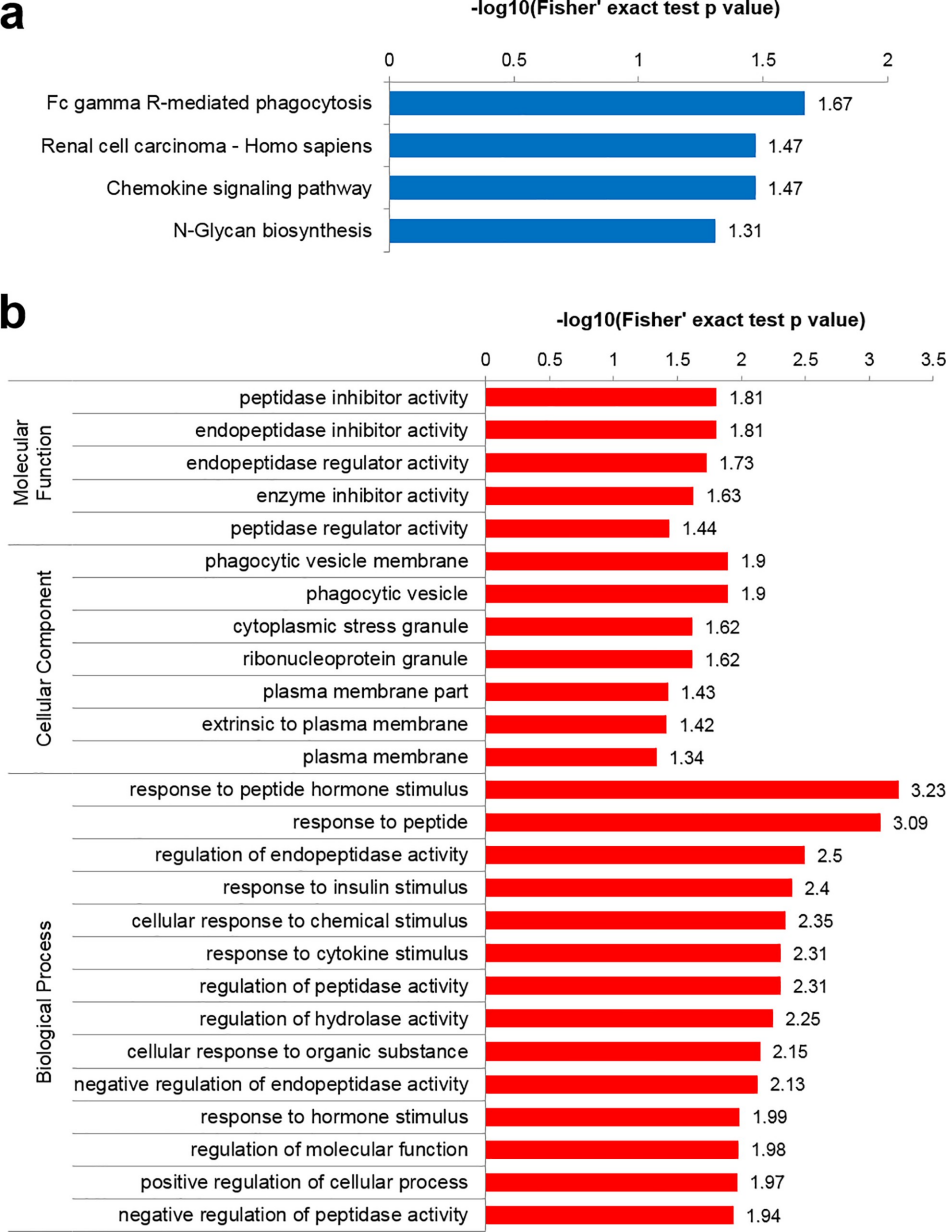
Functional analysis of proteins that were secreted at higher levels by PC3/nKR cells than by PC3 cells. (A) GO-based enrichment analysis and (B) KEGG pathway-based enrichment analysis of the proteins that were secreted at higher levels by PC3/nKR cells than by PC3 cells.

To validate the differential secretion of specific proteins between PC3/nKR and PC3 cells, the concentrated CM samples from the two cell lines were subjected to western blotting with antibodies against the adapter molecule crk, serpin B3, cystatin-M, follistatin, and ULBP2 (**Fig 3**). These five proteins were chosen to validate the quantitative proteomic data because specific antibodies targeting them were commercially available. Cystatin-M was the most highly increased protein in the CM of PC3/nKR cells in comparison with that of PC3 cells (**Table 1**). Serpin B3 was recently reported as a novel candidate biomarker for the diagnosis of bone metastasis in PCa [25], while crk was significantly enriched in three KEGG pathways associated with PCa (**Fig 4A**). Follistatin was not differentially secreted between the two cell lines based on both our proteomic and immunoblot analyses of CM. In our immunoblotting results, the abundance of ULBP2 was reduced in the CM of PC3/nKR cells as compared to that of PC3 cells, which also agreed with the results of our proteomic analysis. Taken together, the immunoblotting results were fully consistent with the proteomic results.

### Global analysis of differentially secreted proteins

**Fig 5:**
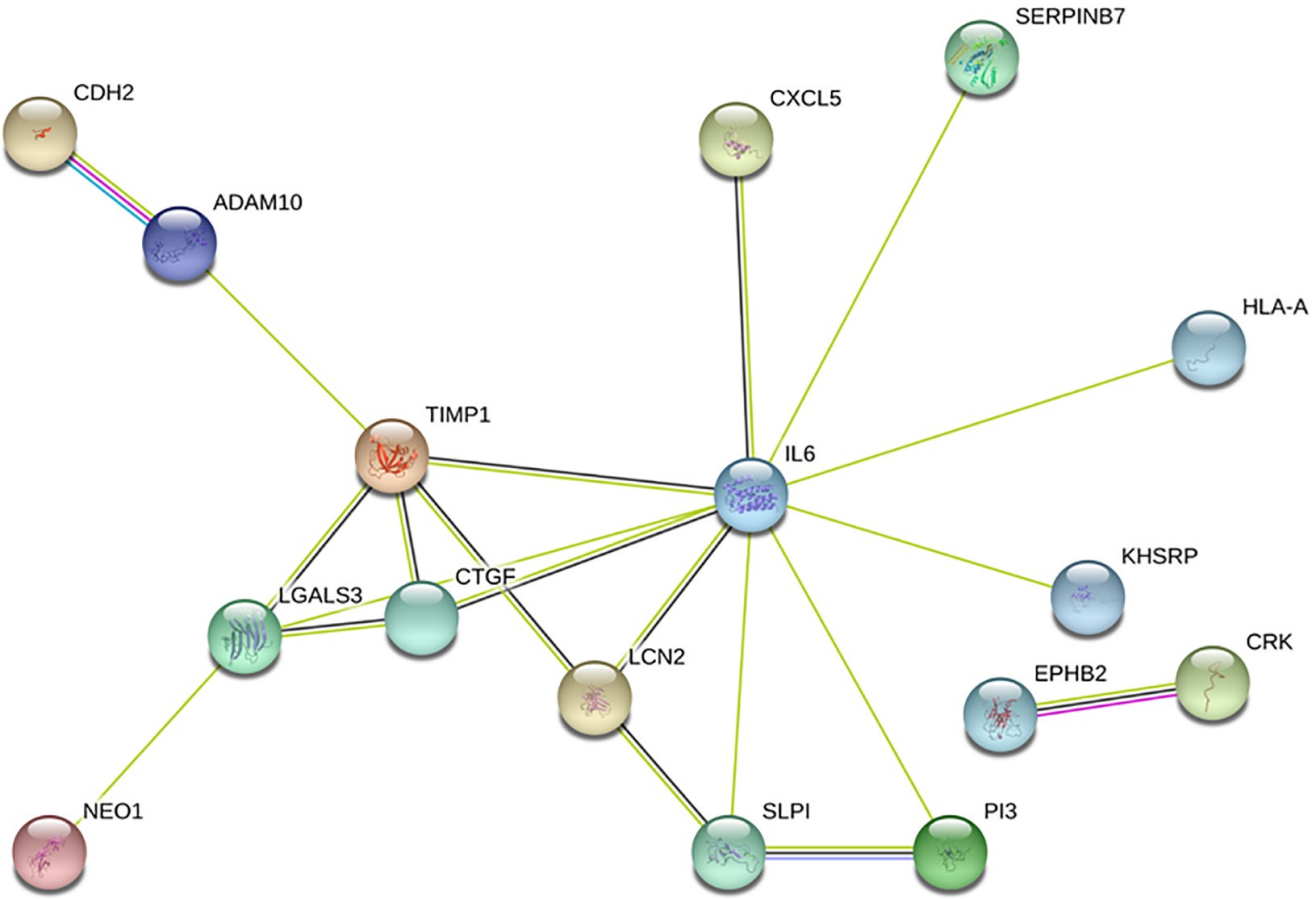
Protein-protein interaction analysis of highly secreted proteins in PC3/nKR cells by STRING.

To further understand the functions of the secreted proteins that we were able to quantify, we performed an enrichment analysis of the GO and KEGG pathways (**Fig 4 and S3 Fig**). Among the 85 differentially secreted proteins, 45 were secreted more and 40 were secreted less by PC3/nKR cells than by PC3 cells. **Table 1** shows information about the former 45 proteins including their database accession number, description, quantitative ratio, and gene name. Based on the enrichment results of the molecular functions category, the proteins that were most highly secreted by PC3/nKR cells in comparison to PC3 cells were related to peptidase and enzyme inhibition activities (**Fig 4B**). Additionally, an enrichment analysis of biological processes revealed that the proteins that were highly secreted by PC3/nKR cells were significantly enriched in secretory peptide functions that include the regulation and responses to peptidase or endopeptidase activities. Meanwhile, the proteins that were secreted at higher levels by PC3 cells in comparison to PC3/nKR cells were enriched in the biological process category of vesicle localization (**S3 Fig**). The KEGG pathway enrichment analysis showed that the proteins that were secreted at higher levels by PC3/nKR cells were related to phagocytosis, renal cell carcinoma, and chemokine signaling pathways (**Fig 4A**). Especially, the adapter molecule crk (protein accession no. P46108) was included in all three of those pathways. The enrichment of secreted proteins assigned to the KEGG pathway of phagocytosis in PC3/nKR cells was consistent with the enrichments in the cellular component categories of phagocytic vesicle membrane and phagocytic vesicle. Based on these analyses, the enrichment in the processes of peptidase activity regulation and phagocytosis might imply that these processes should be the focus of further studies to understand the specific characteristics of PC3/nKR cells. Lastly, we employed the STRING database tool to understand the protein-protein interactions among the secreted proteins that were differentially expressed between the PC3 and PC3/nKR cell lines (**Fig 5**). The 45 proteins that were secreted at higher levels by PC3/nKR cells than by PC3 cells were applied to STRING and unconnected proteins were removed from the results. The STRING results indicated that IL-6 was significantly linked to other over-secreted proteins in PC3/nKR cells. The proteins serpin B7, TIMP1, SLPI, PI3, and IL-6 were included in the GO biological process of negative regulation of endopeptidase and catalytic activity. In addition, these proteins, except for IL-6, were included in the GO molecular function of endopeptidase and/or enzyme inhibitor activity.

## Discussion

In the present study, we investigated the properties of PC3/nKR cells, which were originally isolated from mammary tumor xenografts after implanting PC3 cells into nude mice to study cancer invasion and migration[11]. Furthermore, we analyzed the secreted proteins of PC3/nKR and PC3 cells to understand the molecular mechanisms underlying their phenotypic differences. As shown in Fig 1, the PC3/nKR cells showed a significantly higher NK resistance and migration/invasion activities as compared with PC3 cells. We confirmed that the CM of PC3/nKR contained specific proteins that can stimulate cell migration. We then further analyzed the secretomes of PC3/nKR and PC3 cells using a comparative proteomics approach. In total, we identified 598 proteins that were secreted from both PC3 and PC3/nKR cells, and we were able to quantify 560 of those proteins, among which 45 proteins were secreted more and 40 proteins were secreted less by PC3/nKR cells than by PC3 cells (**Tables 1 and 2**).

First, we observed the NK resistance capacity of PC3/nKR cells (**Fig 1D**). When we separately cultured PC3 and PC3/nKR cells with NK cells, the PC3/nKR cells showed a higher resistance to NK cells than the PC3 cells did. Based on our proteomics results, we identified that HLA-A was secreted more, while NKG2D ligand 2 was secreted less by PC3/nKR cells than by PC3 cells (**Tables 1 and 2**). HLA-A (HLA class I histocompatibility antigen, A-23 α chain) inhibits NK cell receptors to reduce NK cell-induced cytotoxicity [26]. Although it is normally expressed on most healthy cells, its expression is often lost upon malignant transformation or during tumor progression [27]. Therefore, the regulation of HLA I molecules plays a key role in the ability of urologic tumors to escape the immune system [28]. On the contrary, NKG2D (natural-killer receptor group 2, member D) ligand 2 is a well-characterized NK cell-activating receptor that mediates critical signals to render target cells more susceptible to lysis by NK cells [29]. Therefore, a lower expression of NKG2D ligand 2 on the surface of cancer cells favors their survival by reducing their chance of being recognized by NK cells. Many studies have focused on triggering the cell surface expression of NKG2D ligands on tumor cells as a potential therapeutic strategy for PCa [30–32]. Recently, it was reported that the knockdown of IL-6 in castration-resistant PCa cells rendered them more susceptible than control cells to NK cell-mediated cytotoxicity owing to an increased cell surface expression of NKG2D ligands [33]. Our study showed that, similarly to the castration-resistant PCa cells in the previous study, PC3/nKR cells secreted high levels of IL-6 and low levels of NKG2D ligand 2 (Fig 5). The specific secretion of HLA-A, NKG2D ligand 2, and IL-6 by PC3/nKR cells could demonstrate their capacity for resisting NK cell-mediated cytotoxicity.

Furthermore, the migration and invasion activities of PC3/nKR cells were significantly higher than those of PC3 cells, and isolated CM from PC3/nKR cells stimulated the migration and invasion of PC3 cells (Fig 1). These data indicated that the PC3/nKR cells secreted factors that may promote metastatic growth by cell communication within PCa [34]. We focused on 45 highly secreted proteins in the CM of PC3/nKR cells to investigate the metastatic factors stimulating cell migration and invasion. We identified an enrichment of proteins in the categories of regulation of peptidase activity and phagocytosis by GO and KEGG pathway enrichment analyses (**Fig 4**).

Among the highly secreted proteins, crk, serpin B3, and cystatin-M were validated to be highly secreted in PC3/nKR cells by performing western blotting with CM samples from the two cell lines. Especially, the crk adaptor protein was specifically found in three functional KEGG categories where an enrichment of proteins was identified in the CM from PC3/nKR cells, namely Fcγ R-mediated phagocytosis, renal cell carcinoma, and chemokine signaling (**Fig 4A**). The crk is highly expressed in many types of human cancers and may contribute to aggressive cancer phenotypes [35]. The crk protein regulates several signaling cascades involved in cell migration and invasion, and an elevated abundance of crk protein has been reported to be a poor prognostic factor for survival in patients with various cancers [36, 37]. Furthermore, crk protein knockdown was reported to significantly inhibit both migration and invasion in the PCa-like CWR-22rv1 and PC3M cell lines [38, 39]. The high secretion of crk in PC3/nKR cells as compared with PC3 cells might contribute to the enhanced migration and invasion activities of PC3/nKR cells. In the present study, we detected a high secretion of crk in the CM of PC3/nKR cells, while the expression of crk in cell lysates showed no significant difference between the two PCa cell lines (**Fig 3**). Although the molecular functions of secreted crk should be explored in further studies, the high secretion of crk protein by aggressive PCa cells could indicate its potential application as a novel biomarker to predict the risk of metastatic PCa in patients.

In Table 1, the three proteins that showed the largest increase in secretion by PC3/nKR cells as compared with PC3 cells were peptidase inhibitor, cystatin-M, elafin, and neogenin. These proteins were enriched in the GO category of molecular function (**Fig 4B**). Among them, the high secretion of cystatin-M in PC3/nKR cells was confirmed by immunoblotting (**Fig 3**). Cystatin-M/E is a member of a superfamily of evolutionarily-related cysteine protease inhibitors that provide regulatory and protective functions against uncontrolled proteolysis by cysteine proteases [40]. In a previous study, *CST6*, which encodes cystatin-M, was robustly expressed in normal human prostate epithelium, whereas its expression is downregulated in metastatic PCa cell lines and PCa tissues [41]. However, the present result contradicted that previous finding by showing that cystatin-M was present in lysates from both the PCa cell lines examined and was more strongly secreted into the CM of PC3/nKR cells. Although, the exact mechanism was not studied, we propose that different origins of the cell lines might lead to different cystatin-M expression patterns. Further work is needed to elucidate the functional impact of this difference in cystatin-M secretion.

The results of our bioinformatics analyses, including GO enrichment and STRING analyses, indicated that the proteins whose secretion was increased in PC3/nKR cells should influence the regulation and response to peptidase activity. Especially, SERPINB7, TIMP1, SLPI, PI3, and IL-6 were related to the regulation of peptidase activity and interacted with one another. Peptidases can degrade extracellular matrix, which is vital for invasion and metastasis by cancer cells. Previous studies have revealed that both intracellular and extracellular peptidases play roles in several cellular processes including adhesion, angiogenesis, apoptosis, and evasion of the immune system [42–44]. According to recent studies, the overexpression of PI3 in ovarian cancer cells resulted in them gaining resistance against chemotherapeutic drugs-induced apoptosis [45]. SLPI was found to be highly expressed in ovarian cancer cells and its expression may promote cancer progression [46]. In addition, TIMP1 functions in the modulation of extracellular matrix remodeling and its expression is associated with cancer progression [47]. Lastly, SERPINB7 is a peptidase that is well known to be upregulated in patients with breast cancer and lung cancer [48]. Furthermore, both TIMP1 and SLPI are induced by IL-6 as shown by studies on the pathogenesis of liver fibrosis and inflammation caused by innate immunity [49, 50]. Therefore, based on our bioinformatics results, the proteins that were highly secreted by PC3/nKR cells, particularly SERPINB7, TIMP1, SLPI, and PI3, may affect peptidase activity and thereby contribute to the increased migration and invasion of PC3/nKR cells as compared with PC3 cells.

In conclusion, PC3/nKR cells showed high metastatic properties, such as enhanced migration/invasion and increased NK cell resistance as compared with PC3 cells. We demonstrated mechanisms that may contribute to these differences through a comparative secretome analysis of CM samples from PC3 and PC3/nKR cells. PC3/nKR cells showed a decreased secretion of NKG2D ligand 2 and an increased secretion of HLA-A, IL-6, and crk as well as proteins associated with peptidase activity, including SERPINB7, TIMP1, SLPI, and PI3. These secreted factors may contribute to the high migration and invasion activities of PC3/nKR cells. Our findings highlight that PC3/nKR cells represent a new cell system for studying the metastasis and progression of PCa or developing novel anti-tumor or anti-metastatic drugs for the prevention and treatment of aggressive PCa. Although PCa is a curable cancer when it is diagnosed at an early stage [51], many cases end in fatality as a result of metastases from a primary PCa tumor. In the clinical setting, a new method to predict the metastatic potential of PCa is needed, and the secreted proteins discussed in the present study can provide new hints for the development of novel *in vivo* biomarkers that can be measured by analyzing patients’ plasma or tissue samples.

## Supporting information

S1 Table List of all identified protein and peptides in PC-3/nkR and PC-3 cells. (PDF)

S1 Fig Characters of PC3/nKR cells. (A) Cell morphology of PC3 and PC3/nKR (100x). (B) The representative pictures of wound healing assay with PC-3 and PC-3/nKR cells. (C) Natural killer (NK) cell-mediated cytotoxicity assay with PC3 and PC3/nKR. (E) Effects of incubating PC3 cells with the conditioned media (CM) from PC3/nKR cells for 48 h.
(TIF)

S2 Fig Distribution of the TMT ratio of secreted protein in PC-3/nKR and PC-3 cells. (A) The results of SDS gel with Cell Extract (CE) and Conditioned Media (CM) of PC-3 and PC-3/nKR. We confirmed CM of both cell lines are no signal with α-tubulin. (B) Venn diagram of technical replication results using MaxQuant proved 89.2% similarity and (C) the intensity correlation of these data contributed to significance with an R value of 0.9 or higher. (D) The number of quantified proteins were shown. Up or down regulated proteins are searched about 40, 45 proteins respectively.
(TIF)

S3 Fig GO-based enrichment analysis of down-regulated proteins in PC-3/nKR versus PC-3 cells. Vesicle localization of biological process is significantly more enriched in PC-3.
(TIF)
