# Correction: Secretome profiling of PC3/nKR cells, a novel highly migrating prostate cancer subline derived from PC3 cells

**DOI:** 10.1371/journal.pone.0222693

**Published:** 2019-09-13

**Authors:** Ju Mi Jeon, Oh Kwang Kwon, Ann-Yae Na, Eun Ji Sung, Il Je Cho, Mirae Kim, Sung Su Yea, So Young Chun, Jun Nyung Lee, Yun-Sok Ha, Tae Gyun Kwon, Sangkyu Lee

The ninth author’s name is spelled incorrectly. The correct name is: Jun Nyung Lee.

In the Funding Statement, one of the grant numbers from the National Research Foundation of Korea (NRF) grant funded by the Korea government (MSIP) is missing: 2019R1F1A1062343 for SS.
