# Supplementary material for: Secretome profiling of PC3/nKR cells, a novel highly migrating prostate cancer subline derived from PC3 cells
Source: PLoS One. 2019 Aug 12;14(8):e0220807. doi: 10.1371/journal.pone.0220807 (PMC6690527; doi:10.1371/journal.pone.0220807)
Supplement: S1 Table — (PDF) [file pone.0220807.s001.pdf]

Supplementary Table 2: List of all identified protein and peptides in PC-3/nkR and PC-3 cells

Protein ID	Protein names	Gene name	Spectra Count	Unique peptides	Sequence coverage [%]	Detected peptide	128(PC-3nKR) /127(PC-3)	Secreted protein from Only our DB
Q1L6U9	Prostate-associated microseminoprotein	MSMP	21	3	43.2		0.076	+
P05121	Plasminogen activator inhibitor 1	SERPINE1	34	7	22.6		0.217	
E5RHV6	CMP-N-acetylneuraminate-beta-galactosamide-alpha-2,3-sialyltransferase 1	ST3GAL1	3	2	29.9		0.226	
Q5T0D2	UMP-CMP kinase	CMPK1	2	2	7.7	Only 2nd batch	0.232	
P48444	Coatomer subunit delta	ARCN1	2	2	4.5	Only 2nd batch	0.233	
Q06323	Proteasome activator complex subunit 1	PSME1	3	3	15.7		0.266	
Q9HC84	Mucin-5B	MUC5B	7	6	1.3		0.273	
Q16555	Dihydropyrimidinase-related protein 2	DPYSL2	3	3	7		0.295	
O15230	Laminin subunit alpha-5	LAMA5	21	16	6		0.305	
Q9BZM5	NKG2D ligand 2	ULBP2	7	3	12.2		0.319	+
F8W1K5	Protein canopy homolog 2	CNPY2	3	2	25.5	Only 2nd batch	0.335	
P09529	Inhibin beta B chain	INHBB	3	2	5.4		0.362	
Q9H3G5	Probable serine carboxypeptidase CPVL	CPVL	10	3	7.8		0.363	
F8WAE5	Eukaryotic translation initiation factor 2A	EIF2A	2	2	5.9	Only 2nd batch	0.368	
J3QRX2	Granulocyte colony-stimulating factor	CSF3	12	2	7.7		0.370	
Q9UBS4	DnaJ homolog subfamily B member 11	DNAJB11	2	2	6.7		0.372	
P28072	Proteasome subunit beta type-6	PSMB6	3	2	7.9		0.375	
H0YM70	Proteasome activator complex subunit 2	PSME2	4	2	8.8		0.393	+
Q13753	Laminin subunit gamma-2	LAMC2	2	2	2.6		0.395	
Q32MZ4	Leucine-rich repeat flightless-interacting protein 1	LRRFIP1	3	2	3.3	Only 2nd batch	0.403	
P13667	Protein disulfide-isomerase A4	PDIA4	31	12	22.6		0.411	
P27348	14-3-3 protein theta	YWHAQ	4	2	12.7		0.413	
P62805	Histone H4	HIST1H4A	4	2	19.4		0.417	+
Q5T123	SH3 domain-binding glutamic acid-rich-like protein 3	SH3BGRL3	4	2	20.5		0.424	
Q13630	GDP-L-fucose synthase	TSTA3	4	2	9		0.435	+
P04155	Trefoil factor 1	TFF1	21	3	54.8		0.445	
P62937	Peptidyl-prolyl cis-trans isomerase A	PPIA	21	7	40		0.451	
P49006	MARCKS-related protein	MARCKSL1	5	3	33.8		0.452	+
P13693	Translationally-controlled tumor protein	TPT1	15	3	19.8		0.454	
P15018	Leukemia inhibitory factor	LIF	16	2	16.8		0.454	
P12956	X-ray repair cross-complementing protein 6	XRCC6	5	5	11.7		0.459	
P12109	Collagen alpha-1(VI) chain	COL6A1	129	26	33.8		0.461	
P02545	Prelamin-A/C	LMNA	26	13	24.5		0.467	
Q08380	Galectin-3-binding protein	LGALS3BP	12	8	19		0.467	
H0Y704	Zinc finger protein 185	ZNF185	2	2	7.4	Only 2nd batch	0.467	
Q8IWU5	Extracellular sulfatase Sulf-2	SULF2	7	5	6.1		0.467	
Q71UI9	Histone H2A.V	H2AFV	4	2	16.4		0.468	+
Q9NTK5	Obg-like ATPase 1	OLA1	3	2	6.8		0.478	
A0A0A0MSM0	Heat shock protein 105 kDa	HSPH1	4	3	5		0.495	+
P01036	Cystatin-S	CST4	4	2	17.7		0.495	
Q5JWQ6	Neuropilin-1	NRP1	18	6	10.9		0.508	
P37802	Transgelin-2	TAGLN2	24	8	38.7		0.517	
P19875	C-X-C motif chemokine 2	CXCL2	7	2	22.4		0.518	
A0A087X1W2	Protein arginine N-methyltransferase 1	PRMT1	6	4	18.6		0.518	
A0A0A0MRN5	Opioid growth factor receptor	OGFR	2	2	5.4	Only 2nd batch	0.521	+

A0A087WZK9	Eukaryotic translation initiation factor 3 subunit H	EIF3H	3	2	9.2	Only 2nd batch	0.524	+
P23588	Eukaryotic translation initiation factor 4B	EIF4B	2	2	4.3		0.526	+
Q16658	Fascin	FSCN1	17	9	19.3		0.527	
E9PEX6	Dihydrolipoyl dehydrogenase, mitochondrial	DLD	6	2	4.9		0.536	
P61981	14-3-3 protein gamma	YWHAG	7	5	25.5		0.547	
O43291	Kunitz-type protease inhibitor 2	SPINT2	6	2	10.7		0.550	
A6XND1	Insulin-like growth factor-binding protein 3	IGFBP3	15	7	32.3		0.552	
Q99985	Semaphorin-3C	SEMA3C	4	4	5.6		0.553	
M0QYT0	Heterogeneous nuclear ribonucleoprotein L	HNRNPL	5	3	11.8		0.557	+
Q32P28	Prolyl 3-hydroxylase 1	LEPRE1	4	3	5		0.561	
Q9P2E9	Ribosome-binding protein 1	RRBP1	21	11	21.5		0.577	
H0YN18	Proteasome subunit alpha type-4	PSMA4	7	5	19.1		0.580	
P55268	Laminin subunit beta-2	LAMB2	7	6	3.6		0.580	
P04083	Annexin A1	ANXA1	3	3	8.7	Only 2nd batch	0.581	
O60271	C-Jun-amino-terminal kinase-interacting protein 4	SPAG9	2	2	2.6	Only 2nd batch	0.581	
P62826	GTP-binding nuclear protein Ran	RAN	7	4	18.5		0.583	
F8WCZ6	Complement C1s subcomponent	C1S	3	2	5.4		0.587	
Q9Y490	Talin-1	TLN1	6	6	2.8		0.591	
Q09328	Alpha-1,6-mannosylglycoprotein 6-beta-N-acetylglucosaminyltransferase A	MGAT5	6	4	4.6		0.595	+
E9PP21	Cysteine and glycine-rich protein 1	CSRP1	7	3	33.8		0.599	
P02751	Fibronectin	FN1	27	16	9.8		0.603	
I3L3Q4	Glyoxalase domain-containing protein 4	GLOD4	10	5	22		0.612	+
P37837	Transaldolase	TALDO1	9	4	12.8		0.614	
P12814	Alpha-actinin-1	ACTN1	52	13	16.7		0.617	
O00299	Chloride intracellular channel protein 1	CLIC1	6	3	13.7		0.621	
A0A0B4J1Y5	Epidermal growth factor receptor	EGFR	2	2	4.1		0.623	+
E9PQ63	Carbonyl reductase [NADPH] 1	CBR1	6	2	16.9		0.628	
P08133	Annexin A6	ANXA6	4	2	4.3		0.630	
P00736	Complement C1r subcomponent	C1R	8	5	8.1		0.631	
Q9Y617	Phosphoserine aminotransferase	PSAT1	10	7	19.7		0.635	
Q99880	Histone H2B type 1-L	HIST1H2BL	7	3	22.2		0.642	
Q9HAT2	Sialate O-acetylesterase	SIAE	2	2	5	Only 2nd batch	0.649	
F8VPF3	Myosin light polypeptide 6	MYL6	3	2	20		0.651	
E7EUT5	Glyceraldehyde-3-phosphate dehydrogenase	GAPDH	52	7	36.9		0.654	
P13797	Plastin-3	PLS3	17	9	14.1		0.656	
E7EV71	Latent-transforming growth factor beta-binding protein 1	LTBP1	52	19	17.6		0.661	
H3BTN5	Pyruvate kinase	PKM	33	11	24.3		0.665	
P60174	Triosephosphate isomerase	TPI1	54	10	47.2		0.667	
P56537	Eukaryotic translation initiation factor 6	EIF6	4	2	11.4		0.668	
Q9UBR2	Cathepsin Z	CTSZ	28	8	27.7		0.672	
P49721	Proteasome subunit beta type-2	PSMB2	4	2	7.5		0.675	
Q9Y3F4	Serine-threonine kinase receptor-associated protein	STRAP	4	2	6.3		0.675	
P45974	Ubiquitin carboxyl-terminal hydrolase 5	USP5	3	2	2.9		0.676	
P00558	Phosphoglycerate kinase 1	PGK1	49	16	50.6		0.676	
Q9NZ08	Endoplasmic reticulum aminopeptidase 1	ERAP1	15	4	4.6		0.685	
C9JYY6	Neuronal cell adhesion molecule	NRCAM	2	2	1.7	Only 2nd batch	0.686	
Q9GZX9	Twisted gastrulation protein homolog 1	TWSG1	3	2	11.2		0.687	
M0QZK8	Gamma-glutamylcyclotransferase	GGCT	5	2	22.3		0.694	+
F5H7V9	Tenascin	TNC	5	3	2.4		0.697	
Q14974	Importin subunit beta-1	KPNB1	8	5	7.6		0.700	

Q08431	Lactadherin	MFGE8	12	6	18.9		0.701	
P07108	Acyl-CoA-binding protein	DBI	9	2	41.4		0.704	
A0A0C4DFV9	Protein SET	SET	7	5	16.9		0.709	
P31153	S-adenosylmethionine synthase isoform type-2	MAT2A	4	2	7.6		0.712	
P40925	Malate dehydrogenase, cytoplasmic	MDH1	17	8	29.9		0.716	
Q01459	Di-N-acetylchitobiase	CTBS	3	2	7.8		0.717	
Q15582	Transforming growth factor-beta-induced protein ig-h3	TGFBI	14	8	15.1		0.719	
E9PKE3	Heat shock cognate 71 kDa protein	HSPA8	51	12	21.7		0.721	
P06733	Alpha-enolase	ENO1	69	15	37.3		0.721	
P28070	Proteasome subunit beta type-4	PSMB4	10	4	15.9		0.724	
O00410	Importin-5	IPO5	8	4	4		0.725	
Q9BQT9	Calsyntenin-3	CLSTN3	6	4	4.6		0.728	
E7ENU9	Macrophage-capping protein	CAPG	5	3	13		0.731	
Q9Y4K0	Lysyl oxidase homolog 2	LOXL2	23	12	18.6		0.735	
P12004	Proliferating cell nuclear antigen	PCNA	10	2	13		0.735	
H0Y8G5	Heterogeneous nuclear ribonucleoprotein D0	HNRNPD	5	5	19.6		0.737	
P10619	Lysosomal protective protein	CTSA	26	5	11.7		0.738	
P12110	Collagen alpha-2(VI) chain	COL6A2	23	9	8.7		0.741	
Q12792	Twinfilin-1	TWF1	3	2	7.4		0.742	+
J3KTB5	Eukaryotic initiation factor 4A-I	EIF4A1	12	6	28.4		0.743	
P01034	Cystatin-C	CST3	32	5	30.1		0.745	
C9J4N6	Isocitrate dehydrogenase [NADP] cytoplasmic	IDH1	3	2	12.7		0.746	
Q14520	Hyaluronan-binding protein 2	HABP2	3	2	4.1		0.747	
Q02809	Procollagen-lysine,2-oxoglutarate 5-dioxygenase 1	PLOD1	6	6	7.3		0.750	
Q969E4	Transcription elongation factor A protein-like 3	TCEAL3	3	2	12.5		0.752	+
Q9UM22	Mammalian ependymin-related protein 1	EPDR1	5	3	14.7		0.752	
O75509	Tumor necrosis factor receptor superfamily member 21	TNFRSF21	3	2	3.4		0.756	
Q9H1E3	Nuclear ubiquitous casein and cyclin-dependent kinase substrate 1	NUCKS1	12	5	27.2		0.760	
P84090	Enhancer of rudimentary homolog	ERH	5	3	33.7		0.764	+
O15144	Actin-related protein 2/3 complex subunit 2	ARPC2	4	2	6		0.768	
P04792	Heat shock protein beta-1	HSPB1	4	2	10.2		0.769	
P10412	Histone H1.4	HIST1H1E	8	4	14.2		0.773	+
O94760	N(G),N(G)-dimethylarginine dimethylaminohydrolase 1	DDAH1	2	2	4.9	Only 2nd batch	0.773	
Q9BRA2	Thioredoxin domain-containing protein 17	TXNDC17	5	2	18.7		0.774	+
P35237	Serpin B6	SERPINB6	2	2	6.9	Only 2nd batch	0.774	
E9PKW1	Latent-transforming growth factor beta-binding protein 3	LTBP3	8	5	6.8		0.780	
P15144	Aminopeptidase N	ANPEP	3	2	2.5		0.780	
P05556	Integrin beta-1	ITGB1	14	5	7.1		0.782	
P11717	Cation-independent mannose-6-phosphate receptor	IGF2R	8	7	3.3		0.783	
A0A087WWU8		TPM3	25	12	41		0.783	
E2QRB9	Thioredoxin reductase 1, cytoplasmic	TXNRD1	16	7	14.6		0.784	+
Q9BTY2	Plasma alpha-L-fucosidase	FUCA2	7	5	12.6		0.787	
P61088	Ubiquitin-conjugating enzyme E2 N	UBE2N	2	2	13.2		0.790	
P48637	Glutathione synthetase	GSS	3	3	5.5		0.793	
A0A087WY82	Junctional adhesion molecule A	F11R	7	3	15.4		0.796	
P53999	Activated RNA polymerase II transcriptional coactivator p15	SUB1	6	4	34.6		0.798	
P34932	Heat shock 70 kDa protein 4	HSPA4	21	9	16.3		0.798	
P13796	Plastin-2	LCP1	2	2	5.7	Only 2nd batch	0.798	
P40926	Malate dehydrogenase, mitochondrial	MDH2	19	6	21.9		0.799	
K7ELW0	Protein DJ-1	PARK7	26	5	27.8		0.801	

P25788	Proteasome subunit alpha type-3	PSMA3	9	5	16.9		0.805	
P19883	Follistatin	FST	35	11	38.7		0.818	
P17096	High mobility group protein HMG-I/HMG-Y	HMGA1	15	5	48.6		0.825	+
P00492	Hypoxanthine-guanine phosphoribosyltransferase	HPRT1	6	3	17.9		0.826	
Q00610	Clathrin heavy chain 1	CLTC	15	11	8.7		0.831	
Q14914	Prostaglandin reductase 1	PTGR1	2	2	7.3	Only 2nd batch	0.835	
P07195	L-lactate dehydrogenase B chain	LDHB	38	9	24		0.835	
B1AK87	F-actin-capping protein subunit beta	CAPZB	2	2	11.5	Only 2nd batch	0.836	
P31949	Protein S100-A11	S100A11	7	4	31.4		0.837	
P61457	Pterin-4-alpha-carbinolamine dehydratase	PCBD1	6	3	35.6		0.839	+
P26022	Pentraxin-related protein PTX3	PTX3	12	4	16.3		0.839	
P08107	Heat shock 70 kDa protein 1A/1B	HSPA1A	18	8	14.5		0.844	+
Q9UBX1	Cathepsin F	CTSF	3	3	6.8	Only 1st batch	0.844	
P07602	Proactivator polypeptide	PSAP	124	20	41.8		0.847	
Q5SZX9	T-complex protein 1 subunit gamma	CCT3	3	3	13	Only 2nd batch	0.848	
H0YFA9	N-acetylglucosamine-6-sulfatase	GNS	6	4	12.9		0.849	
P04080	Cystatin-B	CSTB	5	2	24.5		0.849	
P09382	Galectin-1	LGALS1	13	5	45.2		0.850	
Q96L35	Ephrin type-B receptor 4	EPHB4	5	4	6.5		0.851	
P20933	N(4)-(beta-N-acetylglucosaminyl)-L-asparaginase	AGA	3	3	10.1		0.853	
P07339	Cathepsin D	CTSD	55	9	27.4		0.856	
O43854	EGF-like repeat and discoidin I-like domain-containing protein 3	EDIL3	26	9	21		0.859	
Q5T7C4	High mobility group protein B1	HMGB1	20	6	30.4		0.860	
Q15149	Plectin	PLEC	29	22	5.2		0.864	
Q06830	Peroxiredoxin-1	PRDX1	34	7	39.2		0.866	
P63104	14-3-3 protein zeta/delta	YWHAZ	15	7	37.1		0.873	
O14745	Na(+)/H(+) exchange regulatory cofactor NHE-RF1	SLC9A3R1	3	2	6.7	Only 2nd batch	0.873	
P10253	Lysosomal alpha-glucosidase	GAA	14	5	5.5		0.875	
P23284	Peptidyl-prolyl cis-trans isomerase B	PPIB	8	5	25.5		0.877	
P25398	40S ribosomal protein S12	RPS12	5	3	22		0.881	
P20962	Parathymosin	PTMS	5	2	22.5		0.882	+
P08670	Vimentin	VIM	20	8	17.4		0.884	
Q01518	Adenylyl cyclase-associated protein 1	CAP1	8	5	10.5		0.885	
O75976	Carboxypeptidase D	CPD	2	2	1.7	Only 2nd batch	0.886	+
Q99519	Sialidase-1	NEU1	5	3	8.2		0.886	+
F8W914	Reticulon-4	RTN4	2	2	7.8	Only 2nd batch	0.889	
P55263	Adenosine kinase	ADK	3	2	8.6		0.892	+
E7EMM4	Acid ceramidase	ASAH1	6	4	9.7		0.893	
P62258	14-3-3 protein epsilon	YWHAE	14	5	17.3		0.894	
E7ET40	Urokinase-type plasminogen activator	PLAU	75	16	40.1		0.894	
A6NKB8	Aminopeptidase B	RNPEP	10	7	16.5		0.895	
P55072	Transitional endoplasmic reticulum ATPase	VCP	50	19	30.8		0.898	
E9PMA0	Apoptosis-inducing factor 1, mitochondrial	AIFM1	2	2	8.8	Only 2nd batch	0.898	
Q96QK1	Vacuolar protein sorting-associated protein 35	VPS35	3	3	5.7	Only 2nd batch	0.898	
P63261	Actin, cytoplasmic 2	ACTG1	78	12	34.7		0.899	
Q32Q12	Nucleoside diphosphate kinase	NME1-NME2	24	7	41.8		0.903	
O00468	Agrin	AGRN	139	36	26.5		0.904	
H0Y2P0	CD44 antigen	CD44	10	4	20.9		0.904	
P19338	Nucleolin	NCL	21	13	13.1		0.907	
D6R9P3	Heterogeneous nuclear ribonucleoprotein A/B	HNRNPAB	3	2	8.2		0.908	

P10909	Clusterin	CLU	27	8	23.4		0.914	
P08581	Hepatocyte growth factor receptor	MET	18	9	8.1		0.914	
P53396	ATP-citrate synthase	ACLY	4	4	4.2		0.916	
P14324	Farnesyl pyrophosphate synthase	FDPS	5	3	9.3		0.919	
P62328	Thymosin beta-4	TMSB4X	7	2	43.2		0.920	
P23526	Adenosylhomocysteinase	AHCY	13	5	10.6		0.921	
H0Y929	PDZ and LIM domain protein 5	PDLIM5	2	2	24.7	Only 1st batch	0.923	
A0A0C4DGZ9	Tripeptidyl-peptidase 1	TPP1	14	6	18.7		0.925	+
P11413	Glucose-6-phosphate 1-dehydrogenase	G6PD	8	5	13.2		0.925	
P62993	Growth factor receptor-bound protein 2	GRB2	3	2	12		0.927	
P21980	Protein-glutamine gamma-glutamyltransferase 2	TGM2	9	5	10.3		0.930	+
Q92692	Poliovirus receptor-related protein 2	PVRL2	3	2	4.3		0.932	
P05362	Intercellular adhesion molecule 1	ICAM1	22	7	16		0.933	
P46940	Ras GTPase-activating-like protein IQGAP1	IQGAP1	5	4	3.3		0.934	
P22314	Ubiquitin-like modifier-activating enzyme 1	UBA1	10	5	6.3		0.935	
F5H365	Protein transport protein Sec23A	SEC23A	3	2	4.6		0.936	
P04075	Fructose-bisphosphate aldolase A	ALDOA	74	17	44		0.937	
H3BVA8	Lysine--tRNA ligase	KARS	4	2	7.8		0.941	+
O60911	Cathepsin L2	CTSL2	3	2	8.7		0.941	
Q71DI3	Histone H3.2	HIST2H3A	4	2	13.2		0.942	+
Q15223	Poliovirus receptor-related protein 1	PVRL1	3	2	3.7		0.944	
P00441	Superoxide dismutase [Cu-Zn]	SOD1	31	7	39		0.944	
P16870	Carboxypeptidase E	CPE	7	4	11.1		0.950	
H3BS10	Beta-hexosaminidase	HEXA	9	5	9.4		0.954	
O75083	WD repeat-containing protein 1	WDR1	10	5	10.6		0.954	
Q9NZV1	Cysteine-rich motor neuron 1 protein	CRIM1	20	10	16.5		0.957	+
P28799	Granulins	GRN	131	25	52.1		0.957	
Q06210	Glutamine--fructose-6-phosphate aminotransferase [isomerizing] 1	GFPT1	3	3	8.3		0.958	+
Q9Y3C6	Peptidyl-prolyl cis-trans isomerase-like 1	PPIL1	3	2	16.3		0.960	
F8VQY6	60S acidic ribosomal protein P0-like	RPLP0	4	2	23.2		0.960	
P17900	Ganglioside GM2 activator	GM2A	13	3	21.2		0.960	
P00390	Glutathione reductase, mitochondrial	GSR	3	2	4.2		0.961	
G3V1A4	Cofilin-1	CFL1	22	7	47		0.961	
P20618	Proteasome subunit beta type-1	PSMB1	8	3	18.7		0.964	
P61158	Actin-related protein 3	ACTR3	8	4	14.6		0.965	
F2Z2W6	Non-histone chromosomal protein HMG-14	HMGN1	3	2	22.7		0.966	+
P61769	Beta-2-microglobulin	B2M	10	4	25.2		0.967	
Q15084	Protein disulfide-isomerase A6	PDIA6	5	3	9.1		0.967	
P68371	Tubulin beta-4B chain	TUBB4B	19	5	14.6		0.967	
Q9Y5S9	RNA-binding protein 8A	RBM8A	2	2	10.9		0.967	
H0Y2X5	Aldehyde dehydrogenase family 1 member A3	ALDH1A3	2	2	2.7		0.968	+
P35241	Radixin	RDX	10	6	7.5		0.970	+
Q99584	Protein S100-A13	S100A13	5	2	20.4		0.970	+
P00338	L-lactate dehydrogenase A chain	LDHA	54	13	32.5		0.970	
P50395	Rab GDP dissociation inhibitor beta	GDI2	22	10	26.5		0.971	
D6REQ6	Ribonuclease T2	RNASET2	5	2	13.3		0.971	
J3KTF8	Rho GDP-dissociation inhibitor 1	ARHGDIA	5	4	20.2		0.971	
P22102	Trifunctional purine biosynthetic protein adenosine-3	GART	6	4	5.4		0.974	
Q14315	Filamin-C	FLNC	21	13	6.7		0.976	
H3BT58	Coactosin-like protein	COTL1	12	3	38.4		0.977	

P12955	Xaa-Pro dipeptidase	PEPD	6	2	6.5		0.978	
P62158	Calmodulin	CALM1	16	8	40.3		0.979	+
Q9UKM7	Endoplasmic reticulum mannosyl-oligosaccharide 1,2-alpha-mannosidase	MAN1B1	2	2	4		0.981	
P54727	UV excision repair protein RAD23 homolog B	RAD23B	9	4	12.2		0.981	
O43707	Alpha-actinin-4	ACTN4	70	27	32.2		0.983	
P48745	Protein NOV homolog	NOV	13	6	18.5		0.986	
P07225	Vitamin K-dependent protein S	PROS1	8	5	10.2		0.988	
P78417	Glutathione S-transferase omega-1	GSTO1	6	4	17		0.988	
P30086	Phosphatidylethanolamine-binding protein 1	PEBP1	10	3	23.5		0.990	
P22626	Heterogeneous nuclear ribonucleoproteins A2/B1	HNRNPA2B1	21	6	18.1		0.991	
O00241	Signal-regulatory protein beta-1	SIRPB1	9	6	16.3		0.992	
C9JXG8	Ran-specific GTPase-activating protein	RANBP1	3	2	20.7		0.993	
Q09028	Histone-binding protein RBBP4	RBBP4	3	3	8.7		0.994	
Q07955	Serine/arginine-rich splicing factor 1	SRSF1	4	3	11.7		0.994	
C9JFR7	Cytochrome c	CYCS	19	3	25.7		0.995	
Q13813	Spectrin alpha chain, non-erythrocytic 1	SPTAN1	18	11	5.7		0.996	+
P07686	Beta-hexosaminidase subunit beta	HEXB	24	9	20.1		0.998	
Q13442	28 kDa heat- and acid-stable phosphoprotein	PDAP1	6	5	32		1.000	
P29317	Ephrin type-A receptor 2	EPHA2	4	3	4		1.006	+
Q9ULF5	Zinc transporter ZIP10	SLC39A10	3	3	4.7		1.006	
O94985	Calsyntenin-1	CLSTN1	60	14	14.6		1.007	
Q96C90	Protein phosphatase 1 regulatory subunit 14B	PPP1R14B	2	2	17		1.007	
E9PSF4	40S ribosomal protein S3	RPS3	2	2	21.4		1.008	
D6RBE9	Annexin	ANXA5	4	2	10.5		1.010	
A2A2V1	Major prion protein	PRNP	9	3	12.4		1.011	
Q7KZF4	Staphylococcal nuclease domain-containing protein 1	SND1	8	3	4.1		1.011	
P14550	Alcohol dehydrogenase [NADP(+)]	AKR1A1	2	2	7.7		1.012	
A0A087X0D5	Pro-cathepsin H	CTSH	3	3	10.2		1.014	
O60568	Procollagen-lysine,2-oxoglutarate 5-dioxygenase 3	PLOD3	9	6	7.6		1.015	
P15311	Ezrin	EZR	26	13	15.9		1.016	
Q99538	Legumain	LGMN	12	6	16.4		1.019	
Q9UHL4	Dipeptidyl peptidase 2	DPP7	6	3	8.1		1.024	
P00491	Purine nucleoside phosphorylase	PNP	15	4	19.4		1.025	
H0YCR7		RNH1	3	3	10.5		1.032	
P09341	Growth-regulated alpha protein	CXCL1	36	4	44.9		1.032	
A6NJA2	Ubiquitin carboxyl-terminal hydrolase	USP14	5	4	10.7		1.032	
P03956	Interstitial collagenase	MMP1	6	3	8.1		1.034	
G5E9W8	Glycogenin-1	GYG1	4	2	10.9		1.039	+
H0YBG7	Heterogeneous nuclear ribonucleoprotein H2	HNRNPH1	3	2	9.7		1.039	
Q8WUM4	Programmed cell death 6-interacting protein	PDCD6IP	6	5	7.1		1.041	
H0Y2Y8	Zyxin	ZYX	3	3	9.3		1.041	
A0A0A0MSA9	Poliovirus receptor	PVR	7	3	8.4		1.047	
P06280	Alpha-galactosidase A	GLA	4	3	8.9		1.047	
P67809	Nuclease-sensitive element-binding protein 1	YBX1	8	3	21		1.047	
Q92626	Peroxidasin homolog	PXDN	11	5	4.5		1.054	
Q29980	MHC class I polypeptide-related sequence B	MICB	2	2	6.5	Only 2nd batch	1.058	+
H3BQZ7	Heterogeneous nuclear ribonucleoprotein U-like protein 2	hCG_2044799	4	3	5		1.060	+
P11021	78 kDa glucose-regulated protein	HSPA5	74	22	39.3		1.061	
P67936	Tropomyosin alpha-4 chain	TPM4	10	4	14.1		1.068	
P50990	T-complex protein 1 subunit theta	CCT8	5	4	9.3		1.070	

P23142	Fibulin-1	FBLN1	11	6	12.9		1.073	
F5H3C5	Superoxide dismutase [Mn], mitochondrial	SOD2	6	4	40.5		1.075	
Q02818	Nucleobindin-1	NUCB1	58	19	43.2		1.082	
F5H6V7	Tumor necrosis factor receptor superfamily member 1A	TNFRSF1A	6	3	15.4		1.083	
P01009	Alpha-1-antitrypsin	SERPINA1	15	7	15.3		1.083	
P48723	Heat shock 70 kDa protein 13	HSPA13	8	5	14.2		1.083	
P28074	Proteasome subunit beta type-5	PSMB5	22	7	27		1.083	
J3KN67		TPM3	3	2	9.1		1.085	
F5GXJ9	CD166 antigen	ALCAM	14	8	15		1.087	
P31431	Syndecan-4	SDC4	21	6	25.3		1.088	+
Q9UKY7	Protein CDV3 homolog	CDV3	8	3	33.7		1.089	
P56159	GDNF family receptor alpha-1	GFRA1	17	7	17.8		1.090	+
E9PQ14	Dipeptidyl peptidase 3	DPP3	5	3	21.5		1.090	
P01137	Transforming growth factor beta-1	TGFB1	6	3	9.5		1.093	
Q12906	Interleukin enhancer-binding factor 3	ILF3	5	3	4		1.093	
O75326	Semaphorin-7A	SEMA7A	17	9	17		1.093	
Q9GZP8	Immortalization up-regulated protein	IMUP	4	2	16		1.095	+
A0A087X253	AP-2 complex subunit beta	AP2B1	6	4	5.3		1.097	+
Q9NP84	Tumor necrosis factor receptor superfamily member 12A	TNFRSF12A	10	2	23.3		1.097	
B8ZZL8	10 kDa heat shock protein, mitochondrial	HSPE1	7	4	27.7		1.101	
P43251	Biotinidase	BTD	5	2	5.7		1.102	
E9PLK3	Puromycin-sensitive aminopeptidase	NPEPPS	11	3	3.7		1.105	
O00754	Lysosomal alpha-mannosidase	MAN2B1	9	5	5.6		1.109	
D6RG15	Twinfilin-2	TWF2	4	3	18.1		1.110	
A0A096LNZ9	Ubiquitin-like protein ISG15	ISG15	4	2	15.4		1.115	+
P26639	Threonine--tRNA ligase, cytoplasmic	TARS	6	4	5.5		1.117	
P28066	Proteasome subunit alpha type-5	PSMA5	10	3	21.2		1.118	
P10124	Serglycin	SRGN	13	2	17.1		1.123	
G3V295	Proteasome subunit alpha type	PSMA6	10	4	16.7		1.128	
Q9UQ80	Proliferation-associated protein 2G4	PA2G4	8	5	16.8		1.131	
Q02790	Peptidyl-prolyl cis-trans isomerase FKBP4	FKBP4	7	4	14.8		1.131	
E9PK47	Phosphorylase	PYGL	5	4	5.5		1.133	
Q86UY0	Thioredoxin domain-containing protein 5	TXNDC5	14	4	13.3		1.134	+
P30530	Tyrosine-protein kinase receptor UFO	AXL	25	5	6.2		1.134	
Q99436	Proteasome subunit beta type-7	PSMB7	7	4	15.9		1.138	
H0YEY4	ADP-sugar pyrophosphatase	NUDT5	3	2	11.7		1.139	
P00918	Carbonic anhydrase 2	CA2	4	3	13.1		1.141	
P39687	Acidic leucine-rich nuclear phosphoprotein 32 family member A	ANP32A	9	3	13.7		1.141	
P26038	Moesin	MSN	14	5	12.1		1.142	
O00151	PDZ and LIM domain protein 1	PDLIM1	7	2	8.5		1.146	
Q16531	DNA damage-binding protein 1	DDB1	7	5	6.1		1.148	
O43396	Thioredoxin-like protein 1	TXNL1	10	4	19.4		1.150	
P13639	Elongation factor 2	EEF2	31	11	15.4		1.151	
Q9NPR2	Semaphorin-4B	SEMA4B	15	5	7.2		1.153	
Q13308	Inactive tyrosine-protein kinase 7	PTK7	4	3	5.1		1.156	
H7BYD9	Peptidyl-glycine alpha-amidating monooxygenase	PAM	5	3	3.5		1.157	
A0A0C4DFS8	Nicotinamide phosphoribosyltransferase	NAMPT	18	6	19		1.158	
E9PNW4	CD59 glycoprotein	CD59	6	2	16.7		1.160	
P30101	Protein disulfide-isomerase A3	PDIA3	39	9	18.6		1.162	
Q14847	LIM and SH3 domain protein 1	LASP1	2	2	6.9	Only 2nd batch	1.164	

A0A087X054	Hypoxia up-regulated protein 1	HYOU1	7	3	4.8		1.165	
P27816	Microtubule-associated protein 4	MAP4	8	7	8.4		1.170	
P29401	Transketolase	TKT	57	17	29.5		1.173	
P07237	Protein disulfide-isomerase	P4HB	28	13	26		1.174	
D6RBD0	Guanine nucleotide-binding protein subunit beta-2-like 1	GNB2L1	3	2	16.2		1.174	
A0A087WYV8	Fibrillin-2	FBN2	15	9	4.8		1.178	
I3L397	Eukaryotic translation initiation factor 5A-1	EIF5A	16	4	24		1.179	
Q9BQE3	Tubulin alpha-1C chain	TUBA1C	20	7	14.9		1.179	
Q9BRK5	45 kDa calcium-binding protein	SDF4	18	7	25.7		1.180	
E5RG62	Putative deoxyribonuclease TATDN1	TATDN1	2	2	19.4	Only 1st batch	1.184	+
P06744	Glucose-6-phosphate isomerase	GPI	25	9	16.5		1.186	
P09467	Fructose-1,6-bisphosphatase 1	FBP1	7	3	11.8		1.188	
P49588	Alanine--tRNA ligase, cytoplasmic	AARS	7	6	6.4		1.189	
Q14126	Desmoglein-2	DSG2	24	6	7.2		1.194	
E9PIA8	Palmitoyl-protein thioesterase 1	PPT1	11	3	18.6		1.194	
Q12841	Follistatin-related protein 1	FSTL1	48	9	27.9		1.195	
Q16706	Alpha-mannosidase 2	MAN2A1	11	8	7.6		1.198	
P98160	Basement membrane-specific heparan sulfate proteoglycan core protein	HSPG2	53	32	9.1		1.200	
A2A2V4	Vascular endothelial growth factor A	VEGFA	9	3	26.4		1.200	
P31939	Bifunctional purine biosynthesis protein PURH	ATIC	2	2	4.7	Only 2nd batch	1.206	
P62633	Cellular nucleic acid-binding protein	CNBP	10	5	33.9		1.206	
D6RIU4	Vesicular integral-membrane protein VIP36	LMAN2	3	2	11.5		1.207	
A0A087WV01	Putative elongation factor 1-alpha-like 3	EEF1A1P5	19	5	12		1.209	
Q8WX77	Insulin-like growth factor-binding protein-like 1	IGFBPL1	4	2	6.1		1.215	
P10599	Thioredoxin	TXN	15	2	21		1.216	
P10809	60 kDa heat shock protein, mitochondrial	HSPD1	10	5	11		1.217	
P35052	Glypican-1	GPC1	21	9	21.3		1.219	+
P05387	60S acidic ribosomal protein P2	RPLP2	9	2	42.6		1.220	
P52907	F-actin-capping protein subunit alpha-1	CAPZA1	6	4	17.1		1.224	
F8VZJ2	Nascent polypeptide-associated complex subunit alpha	NACA	7	3	30.9		1.225	
A0A087WVM3	Protein CYR61	CYR61	28	8	23		1.226	+
P18206	Vinculin	VCL	93	33	30.4		1.226	
P08238	Heat shock protein HSP 90-beta	HSP90AB1	123	31	39.6		1.231	
C9JMY1	Insulin-like growth factor-binding protein 2	IGFBP2	11	5	30.9		1.236	
P52209	6-phosphogluconate dehydrogenase, decarboxylating	PGD	19	8	21.7		1.241	
Q92520	Protein FAM3C	FAM3C	12	4	17.2		1.244	
Q99460	26S proteasome non-ATPase regulatory subunit 1	PSMD1	2	2	2.9	Only 2nd batch	1.246	
Q9NRX4	14 kDa phosphohistidine phosphatase	PHPT1	4	2	16		1.248	+
F5GX11	Proteasome subunit alpha type-1	PSMA1	24	8	29.8		1.251	+
P62942	Peptidyl-prolyl cis-trans isomerase FKBP1A	FKBP1A	24	3	39.8		1.253	
P07737	Profilin-1	PFN1	46	8	65		1.255	
P78324	Tyrosine-protein phosphatase non-receptor type substrate 1	SIRPA	5	3	9.5		1.258	
P09874	Poly [ADP-ribose] polymerase 1	PARP1	2	2	3.1	Only 2nd batch	1.259	
E9PGT1	Translin	TSN	2	2	12.6	Only 1st batch	1.261	
Q9ULV4	Coronin-1C	CORO1C	3	2	4.4		1.264	
S4R3Q6	Vacuolar protein sorting-associated protein 26A	VPS26A	4	2	11.5		1.272	
O95274	Ly6/PLAUR domain-containing protein 3	LYPD3	5	4	15.3		1.278	+
P26006	Integrin alpha-3	ITGA3	7	5	4.8		1.281	+
E7ERF2	T-complex protein 1 subunit alpha	TCP1	4	2	4.6		1.282	
P05067	Amyloid beta A4 protein	APP	68	16	25.8		1.283	

P09936	Ubiquitin carboxyl-terminal hydrolase isozyme L1	UCHL1	15	5	28.7		1.283	
A0A0C4DH07	Latent-transforming growth factor beta-binding protein 4	LTBP4	5	4	4		1.284	
P62987	Ubiquitin-60S ribosomal protein L40	UBA52	16	4	32		1.287	+
P07858	Cathepsin B	CTSB	35	9	33.9		1.287	
K7EP46	Thimet oligopeptidase	THOP1	3	2	5.1	Only 2nd batch	1.288	
Q16851	UTP--glucose-1-phosphate uridylyltransferase	UGP2	2	2	4.3		1.293	+
P07711	Cathepsin L1	CTSL1	13	5	17.1		1.299	
A0A087X2B5	Basigin	BSG	6	2	14.5		1.299	
P30041	Peroxiredoxin-6	PRDX6	9	3	15.2		1.302	
P27797	Calreticulin	CALR	50	12	30		1.304	
O75369	Filamin-B	FLNB	83	42	21.5		1.313	
O43583	Density-regulated protein	DENR	3	2	17.2		1.313	
O43768	Alpha-endosulfine	ENSA	3	2	16.5		1.314	
P41091	Eukaryotic translation initiation factor 2 subunit 3	EIF2S3	4	2	7		1.314	
P12277	Creatine kinase B-type	CKB	6	2	8.1		1.322	
G3V4W0	Heterogeneous nuclear ribonucleoproteins C1/C2	HNRNPC	6	4	16.8		1.324	
P02795	Metallothionein-2	MT2A	29	5	67.2		1.326	
P15291	Beta-1,4-galactosyltransferase 1	B4GALT1	2	2	5.3	Only 1st batch	1.333	
Q01082	Spectrin beta chain, non-erythrocytic 1	SPTBN1	4	4	2.5		1.335	
O75487	Glypican-4	GPC4	2	2	5.2		1.344	
Q06481	Amyloid-like protein 2	APLP2	24	9	12.6		1.345	
E9PES6	High mobility group protein B3	HMGB3	3	2	17.6		1.347	
P55060	Exportin-2	CSE1L	7	3	4.3		1.348	
O00115	Deoxyribonuclease-2-alpha	DNASE2	7	3	10.3		1.348	
B8ZZQ6	Prothymosin alpha	PTMA	21	3	14		1.350	
P00751	Complement factor B	CFB	9	5	7.3		1.352	
P07900	Heat shock protein HSP 90-alpha	HSP90AA1	53	14	17.2		1.364	
Q07954	Prolow-density lipoprotein receptor-related protein 1	LRP1	9	8	1.7		1.373	
Q12805	EGF-containing fibulin-like extracellular matrix protein 1	EFEMP1	45	14	34.3		1.374	
O94907	Dickkopf-related protein 1	DKK1	26	8	34.6		1.380	
K7EKH5	Fructose-bisphosphate aldolase	ALDOC	13	2	19.3		1.382	
O43405	Cochlin	COCH	10	6	11.3		1.385	
O76061	Stanniocalcin-2	STC2	13	3	17.5		1.387	
Q96HE7	ERO1-like protein alpha	ERO1L	3	3	9.2		1.390	
P80723	Brain acid soluble protein 1	BASP1	54	11	75.3		1.394	
P09960	Leukotriene A-4 hydrolase	LTA4H	11	6	10.3		1.397	
Q10471	Polypeptide N-acetylgalactosaminyltransferase 2	GALNT2	5	4	6.5		1.398	
P51858	Hepatoma-derived growth factor	HDGF	18	12	55.4		1.398	
O43505	N-acetyllactosaminide beta-1,3-N-acetylglucosaminyltransferase	B3GNT1	4	3	8.2		1.404	
Q9H773	dCTP pyrophosphatase 1	DCTPP1	5	4	22.9		1.411	+
P61978	Heterogeneous nuclear ribonucleoprotein K	HNRNPK	4	4	10.4		1.412	
Q92820	Gamma-glutamyl hydrolase	GGH	28	8	26.1		1.415	
S4R3V8	Lipolysis-stimulated lipoprotein receptor	LSR	4	3	4.3		1.418	
Q8NBJ4	Golgi membrane protein 1	GOLM1	45	15	38.2		1.420	
P78371	T-complex protein 1 subunit beta	CCT2	4	3	7.3		1.432	
P16035	Metalloproteinase inhibitor 2	TIMP2	17	6	19.5		1.438	
P15514	Amphiregulin	AREG	12	5	16.3		1.451	+
E7ETY2	Treacle protein	TCOF1	14	9	6.3		1.459	
Q5HY54	Filamin-A	FLNA	130	51	25.2		1.460	
Q14978	Nucleolar and coiled-body phosphoprotein 1	NOLC1	4	2	3.3		1.462	

G3V0E5	Transferrin receptor protein 1	TFRC	34	11	19.3		1.462	
G3V5D9	DNA-(apurinic or apyrimidinic site) lyase	APEX1	9	4	35.5		1.464	
P11047	Laminin subunit gamma-1	LAMC1	36	14	11.2		1.471	
P49321	Nuclear autoantigenic sperm protein	NASP	7	3	4.4		1.474	
P19367	Hexokinase-1	HK1	3	2	2.4		1.474	
J3KQ45	Trans-Golgi network integral membrane protein 2	TGOLN2	6	4	12.1		1.491	
P18669	Phosphoglycerate mutase 1	PGAM1	31	10	41.7		1.508	
P31948	Stress-induced-phosphoprotein 1	STIP1	4	4	8.3	Only 1st batch	1.513	
Q13185	Chromobox protein homolog 3	CBX3	4	3	13.7	Only 2nd batch	1.513	
P07942	Laminin subunit beta-1	LAMB1	6	4	4		1.515	
I6L8B7	Fatty acid-binding protein, epidermal	FABP5	26	6	54.5		1.520	
Q9C0C4	Semaphorin-4C	SEMA4C	2	2	3	Only 2nd batch	1.528	
C9JBB3	Tissue factor pathway inhibitor	TFPI	3	2	11.1		1.531	+
G3V511	Latent-transforming growth factor beta-binding protein 2	LTBP2	12	8	5.8		1.532	
P10586	Receptor-type tyrosine-protein phosphatase F	PTPRF	11	7	6.6		1.537	
A2A2D0	Stathmin	STMN1	3	3	37.6		1.538	+
O00391	Sulfhydryl oxidase 1	QSOX1	44	16	28.6		1.541	
Q24JP5	Transmembrane protein 132A	TMEM132A	11	8	11.7		1.545	+
O00469	Procollagen-lysine,2-oxoglutarate 5-dioxygenase 2	PLOD2	30	10	16.7		1.545	
O75882	Attractin	ATRN	12	8	6.4		1.545	
Q00839	Heterogeneous nuclear ribonucleoprotein U	HNRNPU	2	2	2.1		1.555	
Q5TG12	Receptor-type tyrosine-protein phosphatase kappa	PTPRK	3	3	3.1	Only 2nd batch	1.556	
P54802	Alpha-N-acetylglucosaminidase	NAGLU	5	4	8.6		1.559	
Q14118	Dystroglycan	DAG1	19	6	10.6		1.567	
Q09666	Neuroblast differentiation-associated protein AHNAK	AHNAK	9	4	3.9		1.567	
P01024	Complement C3	C3	44	22	14.7		1.591	
Q9HBR0	Putative sodium-coupled neutral amino acid transporter 10	SLC38A10	4	2	2.3		1.600	
P13010	X-ray repair cross-complementing protein 5	XRCC5	5	2	3.7		1.600	
P78504	Protein jagged-1	JAG1	8	4	4.5		1.600	
Q86XX4	Extracellular matrix protein FRAS1	FRAS1	5	3	1		1.604	
P26641	Elongation factor 1-gamma	EEF1G	6	2	5.3	Only 2nd batch	1.612	
P49767	Vascular endothelial growth factor C	VEGFC	4	2	4.5		1.620	
O00560	Syntenin-1	SDCBP	6	3	13.1		1.623	
H0Y8E6	DNA replication licensing factor MCM2	MCM2	2	2	2.5	Only 2nd batch	1.626	
P14314	Glucosidase 2 subunit beta	PRKCSH	14	5	13.6		1.635	
P15104	Glutamine synthetase	GLUL	3	2	4.3		1.636	
P53634	Dipeptidyl peptidase 1	CTSC	19	5	10.6		1.650	
H0YMD0	Annexin	ANXA2	33	9	39.2		1.654	
K7ES54	Follistatin-related protein 3	FSTL3	5	4	27.4		1.655	+
Q13162	Peroxiredoxin-4	PRDX4	10	4	21.4		1.655	
P55327	Tumor protein D52	TPD52	4	3	10.7		1.659	
P50454	Serpin H1	SERPINH1	4	3	7.9		1.667	
P13284	Gamma-interferon-inducible lysosomal thiol reductase	IFI30	5	3	13.2		1.675	
P14625	Endoplasmin	HSP90B1	21	10	12.2		1.676	
P22692	Insulin-like growth factor-binding protein 4	IGFBP4	47	9	38.8		1.678	
P26583	High mobility group protein B2	HMGB2	6	4	22.5		1.681	
P01130	Low-density lipoprotein receptor	LDLR	10	7	9.5		1.686	
E9PN89		HSPA8	5	2	3.5		1.699	
O43278	Kunitz-type protease inhibitor 1	SPINT1	11	6	13		1.707	
P47813	Eukaryotic translation initiation factor 1A, X-chromosomal	EIF1AX	5	3	22.9		1.711	+

Q03405	Urokinase plasminogen activator surface receptor	PLAUR	4	4	17		1.720	
P07996	Thrombospondin-1	THBS1	77	26	29.1		1.737	
F5GZS6	4F2 cell-surface antigen heavy chain	SLC3A2	2	2	3.8		1.751	
P06748	Nucleophosmin	NPM1	12	4	13.6		1.755	+
P31947	14-3-3 protein sigma	SFN	7	3	19.8		1.769	
H0Y875	Calumenin	CALU	8	2	15.6		1.773	
P49327	Fatty acid synthase	FASN	5	3	1.3		1.779	
F5GYN4	Ubiquitin thioesterase OTUB1	OTUB1	5	3	17		1.791	
P23246	Splicing factor, proline- and glutamine-rich	SFPQ	5	4	9.5		1.798	
P15529	Membrane cofactor protein	CD46	8	2	6.1		1.806	
E9PQB5	Lysosomal Pro-X carboxypeptidase	PRCP	4	2	13.8		1.850	
P14543	Nidogen-1	NID1	4	3	4.2		1.851	
Q9UNW1	Multiple inositol polyphosphate phosphatase 1	MINPP1	4	3	7.4		1.857	
Q13332	Receptor-type tyrosine-protein phosphatase S	PTPRS	4	4	2.8		1.862	
Q96C19	EF-hand domain-containing protein D2	EFHD2	5	4	19.6		1.867	+
C9JFE4	COP9 signalosome complex subunit 1	GPS1	3	2	6.4		1.890	
A0A087WXM8	Basal cell adhesion molecule	BCAM	2	2	3.6		1.906	
Q02952	A-kinase anchor protein 12	AKAP12	6	5	5.2		1.961	
P11216	Glycogen phosphorylase, brain form	PYGB	2	2	2.8		1.989	
P24592	Insulin-like growth factor-binding protein 6	IGFBP6	33	5	26.2		1.995	
P08476	Inhibin beta A chain	INHBA	42	12	30.8		1.997	
A0A0C4DGH5	Cullin-associated NEDD8-dissociated protein 1	CAND1	2	2	3	Only 2nd batch	2.008	
P17931	Galectin-3	LGALS3	5	3	14.4		2.022	
F8WEX7	Cholinesterase	BCHE	2	2	4		2.062	
F5H6X6	Neutral alpha-glucosidase AB	GANAB	34	11	17.5		2.063	
Q9UNZ2	NSFL1 cofactor p47	NSFL1C	10	7	25.1		2.066	
Q969H8	UPF0556 protein C19orf10	C19orf10	23	3	22		2.069	
P12429	Annexin A3	ANXA3	10	7	27.2		2.076	
P29279	Connective tissue growth factor	CTGF	2	2	6	Only 2nd batch	2.090	
B8ZZU8	Transcription elongation factor B polypeptide 2	TCEB2	3	2	10.6		2.116	+
Q9BYC5	Alpha-(1,6)-fucosyltransferase	FUT8	4	2	4		2.134	+
K7EN15	Soluble calcium-activated nucleotidase 1	CANT1	5	3	8.7		2.149	+
A0A087WZH7	Myristoylated alanine-rich C-kinase substrate	MARCKS	7	6	33.9		2.252	+
P04062	Glucosylceramidase	GBA	8	6	11.6		2.261	
Q92945	Far upstream element-binding protein 2	KHSRP	5	2	6.8		2.272	
P19022	Cadherin-2	CDH2	12	3	6.3		2.396	
P42830	C-X-C motif chemokine 5	CXCL5	8	4	36.8		2.396	
Q5H9A7	Metalloproteinase inhibitor 1	TIMP1	44	6	54.5		2.428	
P30447	HLA class I histocompatibility antigen, A-23 alpha chain	HLA-A	13	6	21.6		2.436	+
D3DQB3	Testican-1	SPOCK1	3	2	9.5		2.477	+
Q8NC51	Plasminogen activator inhibitor 1 RNA-binding protein	SERBP1	9	5	16.9		2.538	
P17174	Aspartate aminotransferase, cytoplasmic	GOT1	10	7	18.6		2.571	
C9J5B0	Interleukin-6	IL6	10	4	29.6		2.645	
O75635	Serpin B7	SERPINB7	6	4	10.5		2.648	+
P46108	Adapter molecule crk	CRK	2	2	6.9	Only 2nd batch	2.748	+
P43121	Cell surface glycoprotein MUC18	MCAM	4	2	3.9		2.975	
G3V2V8	Epididymal secretory protein E1	NPC2	34	6	54.9		3.056	
K7EL68	Hsp90 co-chaperone Cdc37	CDC37	6	3	17.9		3.107	+
Q13200	26S proteasome non-ATPase regulatory subunit 2	PSMD2	3	2	4.2		3.111	
B1AKC9	Ephrin type-B receptor 2	EPHB2	5	4	5.9		3.192	

Q15459	Splicing factor 3A subunit 1	SF3A1	3	2	3.3	Only 2nd batch	3.195	
P05198	Eukaryotic translation initiation factor 2 subunit 1	EIF2S1	4	2	8.6		3.341	
Q99674	Cell growth regulator with EF hand domain protein 1	CGREF1	7	5	22.6		3.364	
Q8NBP7	Proprotein convertase subtilisin/kexin type 9	PCSK9	4	2	3.3		3.376	
Q92876	Kallikrein-6	KLK6	2	2	16.4	Only 2nd batch	3.521	
Q8WW12	PEST proteolytic signal-containing nuclear protein	PCNP	2	2	17.4		3.642	+
P03973	Antileukoproteinase	SLPI	11	3	17.4		3.957	
O14672	Disintegrin and metalloproteinase domain-containing protein 10	ADAM10	5	3	6.1		4.439	
Q5K684	Serpin B3	SERPINB3	9	3	7.7		4.533	+
Q9GZL7	Ribosome biogenesis protein WDR12	WDR12	3	3	10.2		4.738	
E9PKG2	Low-density lipoprotein receptor-related protein 8	LRP8	3	2	5.6		4.741	+
B7Z5J4	Carboxypeptidase A4	CPA4	2	2	6	Only 2nd batch	4.769	
P80188	Neutrophil gelatinase-associated lipocalin	LCN2	27	5	36.9		5.073	
Q92859	Neogenin	NEO1	3	2	2.1		5.278	
P19957	Elafin	PI3	10	3	25.6		5.627	
Q15828	Cystatin-M	CST6	3	2	21.5		8.690	
P08243	Asparagine synthetase [glutamine-hydrolyzing]	ASNS	3	2	4.3		-	+
Q9Y266	Nuclear migration protein nudC	NUDC	3	3	10		-	+
Q9NX62	Inositol monophosphatase 3	IMPAD1	2	2	4.2	Only 2nd batch	-	+
Q8NFZ8	Cell adhesion molecule 4	CADM4	3	2	6.4		-	+
O76003	Glutaredoxin-3	GLRX3	2	2	6.9		-	+
Q9HAV7	GrpE protein homolog 1, mitochondrial	GRPEL1	3	2	12.4		-	+
Q7LGC8	Carbohydrate sulfotransferase 3	CHST3	2	2	6.5	Only 2nd batch	-	+
K7EPL2	SUMO-activating enzyme subunit 2	UBA2	3	2	9.2		-	+
E9PLA9	Caprin-1	CAPRIN1	2	2	12.4		-	+
Q14703	Membrane-bound transcription factor site-1 protease	MBTPS1	2	2	2.9		-	+
Q9P258	Protein RCC2	RCC2	2	2	5.9	Only 2nd batch	-	+
B4DLR8	NAD(P)H dehydrogenase [quinone] 1	NQO1	3	2	11.9		-	+
Q96FJ2	Dynein light chain 2, cytoplasmic	DYNLL2	2	2	20.2	Only 2nd batch	-	+
A8MXH2	Nucleosome assembly protein 1-like 4	NAP1L4	2	2	13.5	Only 2nd batch	-	
Q15393	Splicing factor 3B subunit 3	SF3B3	3	3	3.7		-	
Q86SQ4	G-protein coupled receptor 126	GPR126	4	2	2.4		-	
X1WI29	Laminin subunit beta-3	LAMB3	2	2	21.4	Only 1st batch	-	
Q8NCW5	NAD(P)H-hydrate epimerase	APOA1BP	2	2	9	Only 1st batch	-	
Q9Y265	RuvB-like 1	RUVBL1	2	2	4.2	Only 1st batch	-	
F5H7V1	Nardilysin	NRD1	2	2	2.5	Only 2nd batch	-	
H3BPC4	SUMO-conjugating enzyme UBC9	UBE2I	2	2	30	Only 2nd batch	-	
O15067	Phosphoribosylformylglycinamidine synthase	PFAS	2	2	1.3	Only 2nd batch	-	
P34096	Ribonuclease 4	RNASE4	2	2	16.3	Only 2nd batch	-	
Q16610	Extracellular matrix protein 1	ECM1	7	5	12.6		-	
F8W6I7	Heterogeneous nuclear ribonucleoprotein A1	HNRNPA1	5	4	12.7		-	
P36871	Phosphoglucomutase-1	PGM1	4	3	7.3		-	
Q9Y230	RuvB-like 2	RUVBL2	4	3	7.6		-	
P52823	Stanniocalcin-1	STC1	3	3	14.2		-	
H3BM42	Golgi apparatus protein 1	GLG1	6	2	3.9		-	
H0Y586	Proteasome subunit alpha type-7	PSMA7	3	2	12.8		-	
F8WC54	Disintegrin and metalloproteinase domain-containing protein 9	ADAM9	3	2	3.8		-	
O14980	Exportin-1	XPO1	3	2	2		-	
A6QRJ1	V-type proton ATPase subunit S1	ATP6AP1	3	2	13		-	
P23381	Tryptophan--tRNA ligase, cytoplasmic	WARS	2	2	5.3		-	

P55884	Eukaryotic translation initiation factor 3 subunit B	EIF3B	2	2	2.8		-	
Q92973	Transportin-1	TNPO1	2	2	3.7		-	
E9PMD7	Serine/threonine-protein phosphatase	PPP1CA	2	2	7.1	Only 2nd batch	-	
