# Supplementary figures and images for: Secretome profiling of PC3/nKR cells, a novel highly migrating prostate cancer subline derived from PC3 cells
Source: PLoS One. 2019 Aug 12;14(8):e0220807. doi: 10.1371/journal.pone.0220807 (PMC6690527; doi:10.1371/journal.pone.0220807)

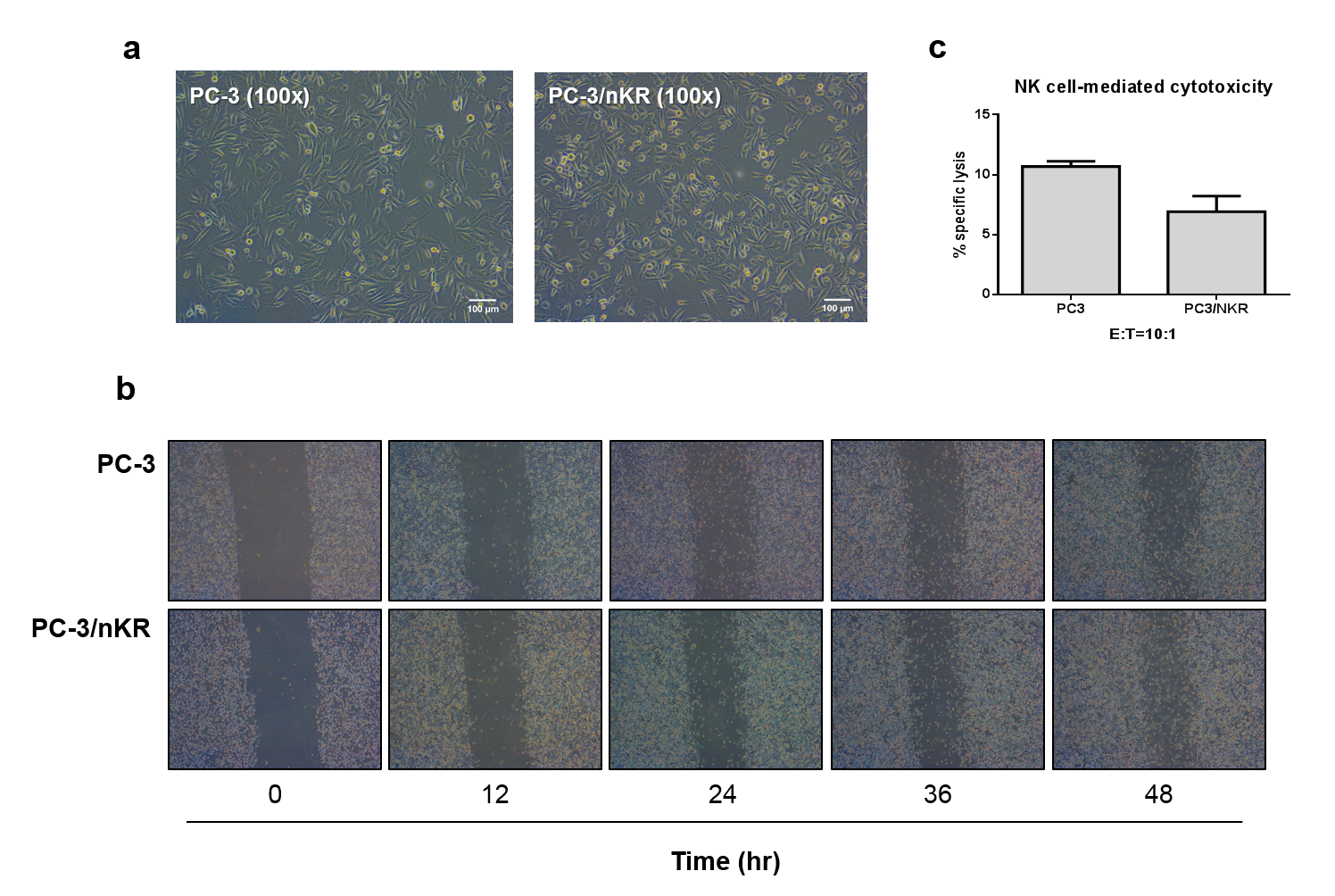

Supplement: S1 Fig — (A) Cell morphology of PC3 and PC3/nKR (100x). (B) The representative pictures of wound healing assay with PC-3 and PC-3/nKR cells. (C) Natural killer (NK) cell-mediated cytotoxicity assay with PC3 and PC3/nKR. (E) Effects of incubating PC3 cells with the conditioned media (CM) from PC3/nKR cells for 48 h. (TIF) [file pone.0220807.s002.tif]

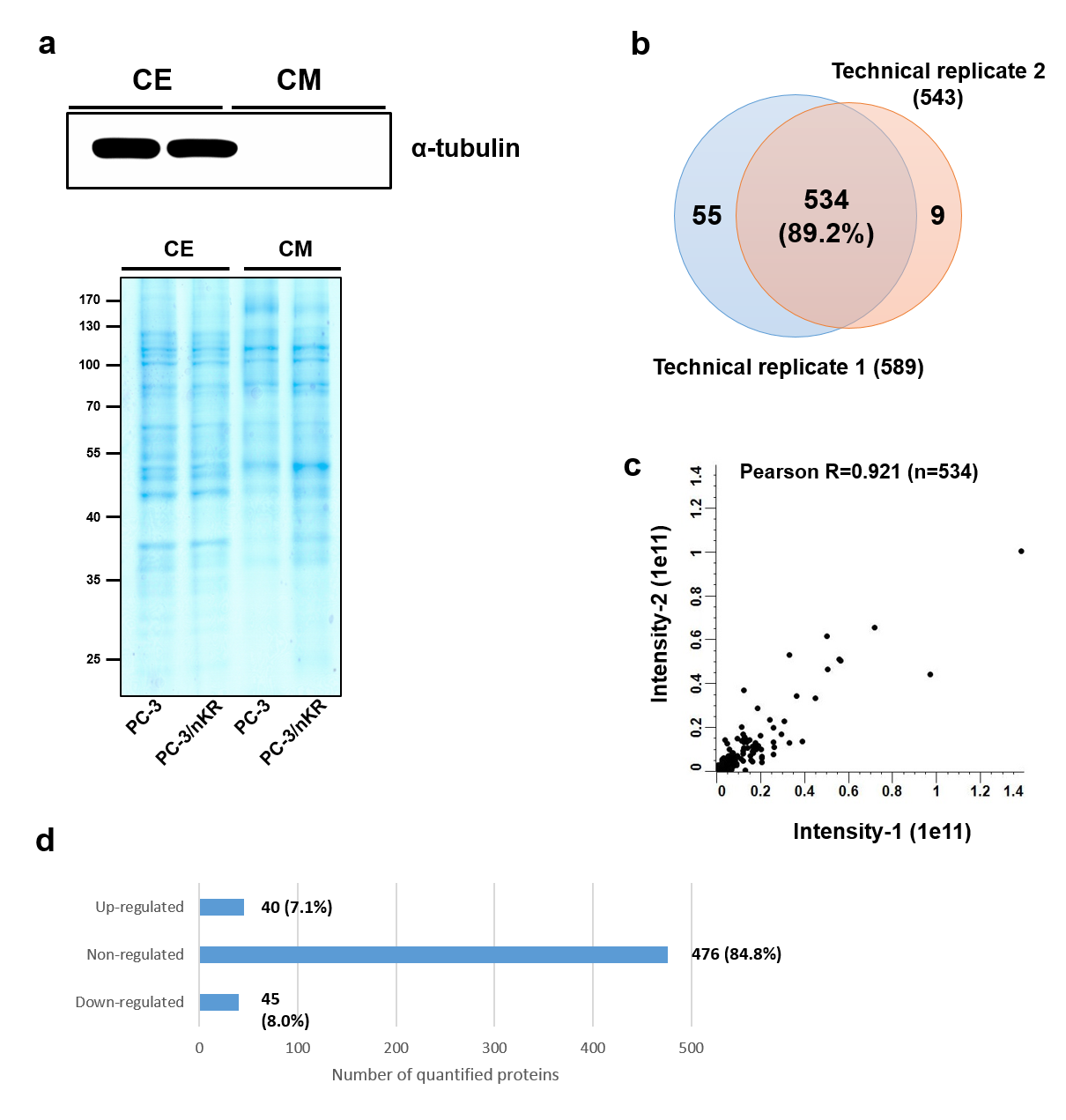

Supplement: S2 Fig — (A) The results of SDS gel with Cell Extract (CE) and Conditioned Media (CM) of PC-3 and PC-3/nKR. We confirmed CM of both cell lines are no signal with α-tubulin. (B) Venn diagram of technical replication results using MaxQuant proved 89.2% similarity and (C) the intensity correlation of these data contributed to significance with an R value of 0.9 or higher. (D) The number of quantified proteins were shown. Up or down regulated proteins are searched about 40, 45 proteins respectively. (TIF) [file pone.0220807.s003.tif]

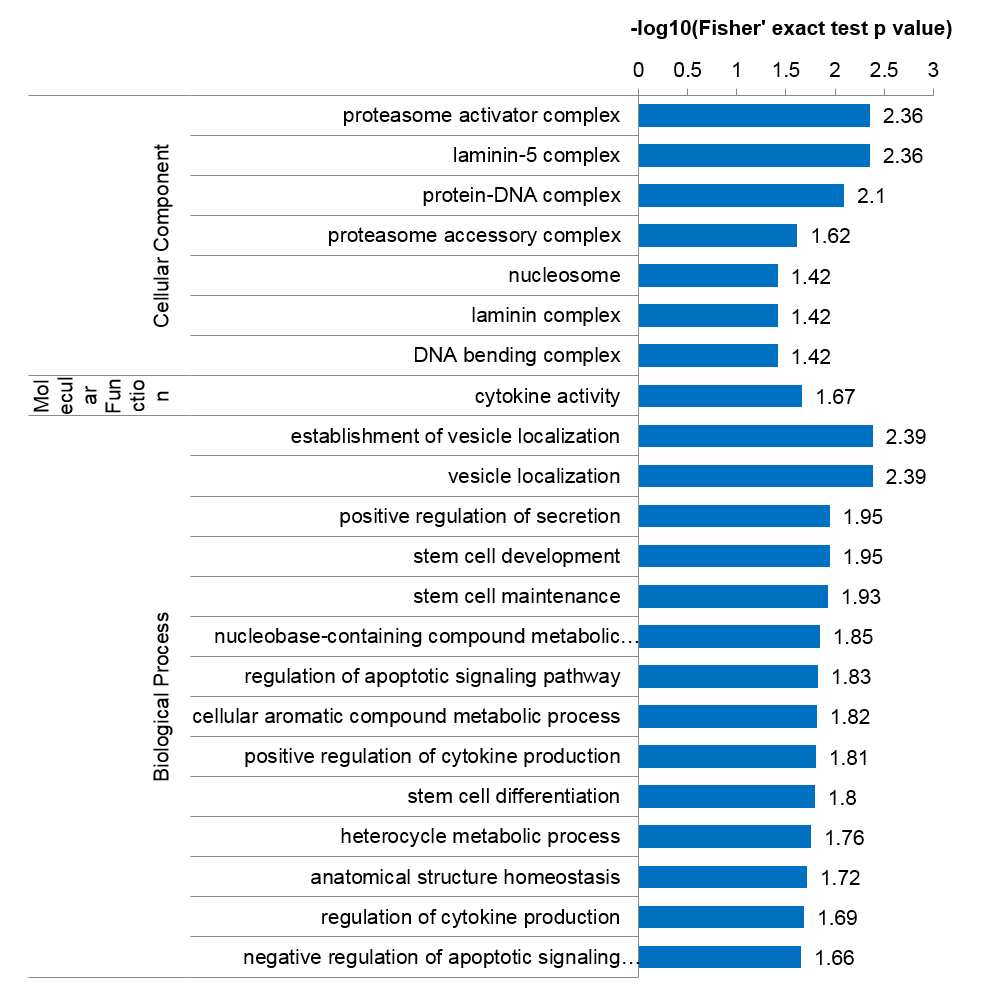

Supplement: S3 Fig — Vesicle localization of biological process is significantly more enriched in PC-3. (TIF) [file pone.0220807.s004.tif]
